# Harnessing Fc/FcRn Affinity Data from Patents with Different Machine Learning Methods

**DOI:** 10.3390/ijms24065724

**Published:** 2023-03-16

**Authors:** Christophe Dumet, Martine Pugnière, Corinne Henriquet, Valérie Gouilleux-Gruart, Anne Poupon, Hervé Watier

**Affiliations:** 1 EA7501, Université de Tours, 37041 Tours, France; 2 MAbSilico, 1 Impasse du Palais, 37000 Tours, France; 3 Institut de Recherche en Cancérologie de Montpellier, Université de Montpellier, 34090 Montpellier, France; 4 Laboratoire d’Immunologie, Centre Hospitalier Universitaire, 37044 Tours, France; 5 Physiologie de la Reproduction et des Comportements, INRAE UMR-0085, CNRS UMR-7247, Université de Tours, 37380 Nouzilly, France; 6 Musca, Inria Saclay-Île-de-France, 91120 Palaiseau, France

**Keywords:** FcRn, antibody, Fc variant, machine learning

## Abstract

Monoclonal antibodies are biopharmaceuticals with a very long half-life due to the binding of their Fc portion to the neonatal receptor (FcRn), a pharmacokinetic property that can be further improved through engineering of the Fc portion, as demonstrated by the approval of several new drugs. Many Fc variants with increased binding to FcRn have been found using different methods, such as structure-guided design, random mutagenesis, or a combination of both, and are described in the literature as well as in patents. Our hypothesis is that this material could be subjected to a machine learning approach in order to generate new variants with similar properties. We therefore compiled 1323 Fc variants affecting the affinity for FcRn, which were disclosed in twenty patents. These data were used to train several algorithms, with two different models, in order to predict the affinity for FcRn of new randomly generated Fc variants. To determine which algorithm was the most robust, we first assessed the correlation between measured and predicted affinity in a 10-fold cross-validation test. We then generated variants by in silico random mutagenesis and compared the prediction made by the different algorithms. As a final validation, we produced variants, not described in any patents, and compared the predicted affinity with the experimental binding affinities measured by surface plasmon resonance (SPR). The best mean absolute error (MAE) between predicted and experimental values was obtained with a support vector regressor (SVR) using six features and trained on 1251 examples. With this setting, the error on the log(K_D_) was less than 0.17. The obtained results show that such an approach could be used to find new variants with better half-life properties that are different from those already extensively used in therapeutic antibody development.

## 1. Introduction

The wide therapeutic success of monoclonal antibodies (mAbs) in numerous indications is mainly due to their high target specificity and their long half-life, ranging from 3 days to more than 30 days for non-engineered mAbs. Further enhancing the half-life of therapeutic antibodies allows a decrease in the periodicity of administration and increases their efficacy [1,2,3]. Antibody half-life depends on many factors, such as the target, target-mediated drug disposition [4], heavy-chain allotype [5,6], and presence of anti-drug Abs. However, the predominant mechanism determining the half-life is the binding of the IgG Fc portion to FcRn, which protects IgG from catabolism. This binding is pH-dependent due to the presence of histidine residues in the Fc portion and glutamic acid residues in FcRn. The high-affinity complex is formed in endosomal compartments at low pH (pH 6) but not extracellularly at physiological pH (pH 7.4). In order to harness this mechanism, many companies have tested Fc mutations improving the binding to FcRn at acidic pH only, which improves the endosomal recycling efficiency and enhances the pharmacokinetics of the antibody. For example, Medimmune and Xencor have patented the M252Y/S254T/T256E and M428L/N434S mutations, respectively [1,7,8]. Finding useful mutations is not trivial, since increasing binding at acidic pH often results in a simultaneous increase in affinity at neutral pH, which mitigates the desired effect [9]. Such mutations can even worsen the pharmacokinetic properties [7,10] because of reduced antibody release from FcRn back to the plasma. In contrast, some companies voluntarily enhance the binding to FcRn at neutral pH in order to flush out antigens more rapidly [9,11].

To find the right mutants, alanine scanning combined with rational design was initially the most commonly used technique [12], leading to the identification of amino acids that are essential for the binding of Fc to FcRn. For example, mutation of the isoleucine at position 253 [12] or histidine at position 310 [13] by any other amino acid diminishes or abrogates the binding. Conversely, substitution of asparagine at position 434 by a hydrophobic amino acid (N434A, N434W, N434Y, N434F) or other types of amino acids (N434H, N434G, N434S, N434Q) [9,14] enhances the binding. More powerful approaches were then developed to find new variants, such as phage display [9], random plus directed mutagenesis [15], or combinations of in silico methods and rational design [16,17]. However, the generated mutants frequently appear as a combination of already described single mutations. Moreover, these methods still require experimental testing of many variants because of their low performance in predicting the combinatorial effect of several single mutations.

Several in silico methods have been developed to predict protein/protein binding affinity [18]. These methods are generally pre-determined equations (scoring functions) of energy terms, and the weights of the terms are optimized by machine learning on experimental datasets comprising various protein–protein complex structures. If these methods perform well with the training dataset, they generally show low correlation with a new test set, which is certainly due to the fact that the test set diverges too much from the learning set [19,20]. Indeed, as with all machine learning settings, the final performance is highly dependent on the quality and diversity of the learning dataset. Algorithms dedicated to the prediction of Fc/FcRn binding affinity have been developed [21,22]. However, the precision of these scoring functions is low, especially for evaluating the impact of multiple mutations. Most of these algorithms suffer from too reduced learning sets. Nevertheless, a lot of data are available regarding Fc/FcRn variants, but they have not been exploited with these methods yet. Indeed, only a selection of variants is usually described in the scientific literature, even in supplementary data, although a larger number of tested variants can be retrieved from patent applications or patents. For example, researchers from Chugai Pharma tested more than 1000 variants, but the comprehensive set of mutated variants can only be found in some patent applications (e.g., WO2013046704), whereas only 7 variants are described in the corresponding article [23].

In the present work, we collected these data in order to constitute a specific Fc/FcRn dataset that could be used in machine learning algorithms. Our dataset of Fc variants was mainly collected from the patent literature. We then trained different algorithms with Fc/FcRn parameters calculated with bioinformatic tools, together with affinity data, and assessed the performance of the different algorithms in a 10-fold cross-validation setting. We also evaluated the algorithms by comparing the distribution of predicted affinities for thousands of in silico randomly generated Fc variants. Finally, to validate the robustness of the models, we produced three new variants with three, five, and seven mutations and compared the predicted affinity with the experimental binding affinities measured by SPR.

## 2. Results

### 2.1. Description of the Fc Variant Dataset and Creation of the Learning Sets

Global patent database software was queried with various keywords, such as FcRn, antibodies, variant, mutation, or half-life, in the patents claims to specifically retrieve FcRn-directed antibody-engineering-related documents. This request resulted in 225 documents (patents or patent applications), which were analyzed in order to eliminate documents that did not contain relevant examples, or that contained only variants with no amino acid substitution directly in the interface of the Fc/FcRn complex. As of December 2020, the dataset contained 1323 variants from 20 patents. Among them, 1099 are variants with an affinity reported at pH 7.0 only, measured with an accurate technique (SPR), with the same protocol (T = 25 °C, same buffer and procedures), and by the same company. The 224 other variants are reported at pH 6.0 only, measured by ELISA or Amplified Luminescent Proximity Homogeneous Assay (temperature unknown, reported as room temperature). The Fc variants (mainly IgG1) of the dataset can have up to 12 mutations. In this study, we built two learning sets of different sizes. The first learning set (FLS) contains very homogeneous data: the 1099 Fc variants evaluated at pH 7.0 by SPR with the same protocol. The second learning set (SLS) also contains the 224 variants only evaluated at pH 6.0 in addition to the 1099 variants of the FLS. The contents of the two datasets are summarized in Table 1. In an attempt to use all the available data, despite the pH difference, we homogenized the data by multiplying by 68 the K_D_ of the 224 examples reported at pH 6.0 only, since the wild-type Fc was reported to have a K_D_ of 1.3 × 10^−6^ M at pH 6.0 and 8.8 × 10^−5^ M at pH 7.0. Indeed, it has already been proposed by other authors that the variation in the log(K_D_) with the pH was fairly linear between pH 6.0 and 7.4 [24]. The relevance of this first approach will be further discussed in the discussion section.

### 2.2. Algorithms and Tested Features

The 3D structure of the 1323 Fc variants were modeled from the Fc/FcRn co-crystal (4N0U.pdb file [13]) with PyMOL v2.5.4, and features reported to be relevant in previous studies [25,26,27] were calculated with the CCP4 software v8.0.009. In total, 147 features were initially considered (Table A1) and collected from the 1323 Fc/FcRn 3D models. In our model, variants considered as different in the original patent can lead to duplicates since not all amino acids are used for computing parameters. For example, if a variant has the S239K/T256E substitution and the other variant has the L235R/T256E substitution, it is considered as a duplicate because the influence of the S239K or L235R substitutions is ignored in our model. Including these positions in the study was nevertheless considered. However, from our dataset, mutations at these two positions do not significantly alter the affinity. Consequently, in this example, only the T256E substitution is taken into account, and the two variants appear as duplicates in our set. We thus eliminated such duplicates, which could bias the training results. As a result, the FLS contains 1048 examples and the SLS 1251 examples.

We then tested different machine learning (ML) algorithms using the FLS and SLS learning sets. Among the scikit-learn library [28], we chose four different algorithms: support vector regressor (SVR), multi-linear regression (MLR), multi-layer perceptron (MLP), and random forest regressor (RFR). These methods were well suited for the type of data we had and the type of predictions we wanted to obtain. Moreover, they are quite simple in their principles, and we wanted to see if the parameters we had in mind were sufficient for the task. Using complex and more opaque artificial intelligence methods hinders problems such as insufficient examples in the learning set or overfitting.

We first used the SelectFromModel method of scikit-learn. This method evaluates the importance of each parameter based on the optimized models. The parameter with the lowest importance is removed, and the performance of the new model is computed. If the performance is not altered, the removal is confirmed, and removal of the next lowest importance parameter is evaluated. The iteration stops when the performance as compared to the initial model is altered by removal of the lowest importance parameter. Application to our two models consistently retained 25 to 28 features for the FLS and 10 to 12 features for the SLS. This first reduction in the number of features greatly improved the performance (evaluated by 10-fold cross-validation) of the MLR algorithm. The performance of SVR, RFR, and MLP remained unchanged (data not shown), but with a net gain in calculation speed. 

**Figure 1:**
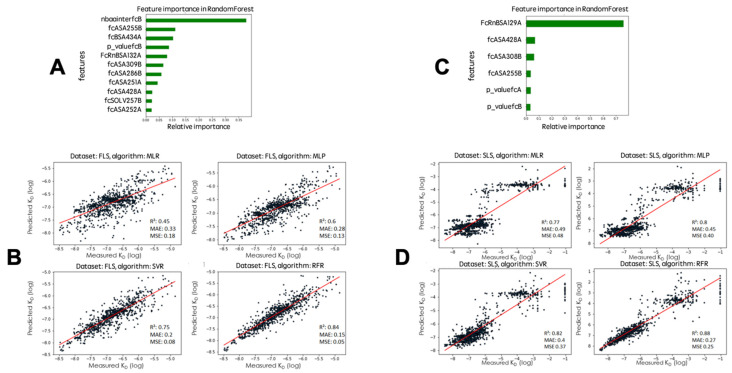
Parameter selection and machine learning performance; parameters are defined in Table A1. (**A**) Impact of the features in the FLS model. (**B**) Scatterplots of the 10-fold cross-validation predictions with the 4 algorithms trained on FLS. (**C**) Impact of the features in the SLS model. (**D**) Scatterplots of the 10-fold cross-validation predictions with the 4 algorithms trained on SLS. The scatterplots show the experimental (X axis) vs predicted (Y axis) affinities of the variants. Regression line is in red; R^2^: coefficient of determination; MAE: mean absolute error; MSE: mean squared error; the features kept for the models have been evaluated with the “SelectFromModel” of scikit-learn.

We then removed features that were highly correlated (evaluated by the pandas.DataFrame.corr method) and kept 11 features for the FLS and 6 for the SLS (Figure 1). This second step slightly improved the performance of the MLR with the FLS and slightly decreased the performance of the other algorithms with the SLS. However, this further dimension reduction is useful to prevent overfitting. Further dimension reduction (removing of features) negatively impacted the performance of all algorithms. 

We compared the results obtained for the two learning sets using the optimal number of features: FLS with 11 features and SLS with 6 features. The most important feature of the FLS model (35% relative importance) is the number of atoms interacting between the β chain of FcRn (β2-microglobulin) and the Fc (Figure 1). The accessible surface area of residue at position 255 and buried surface area of residue at position 434 of the Fc come in second and third position, respectively (Figure 1). The other features have lower impact but altogether account for about half of the model information (Figure 1). The most important feature retained with the SLS model is the buried surface area of the amino acid at position 129 of FcRn, with a relative importance of 0.7.

**Figure 2:**
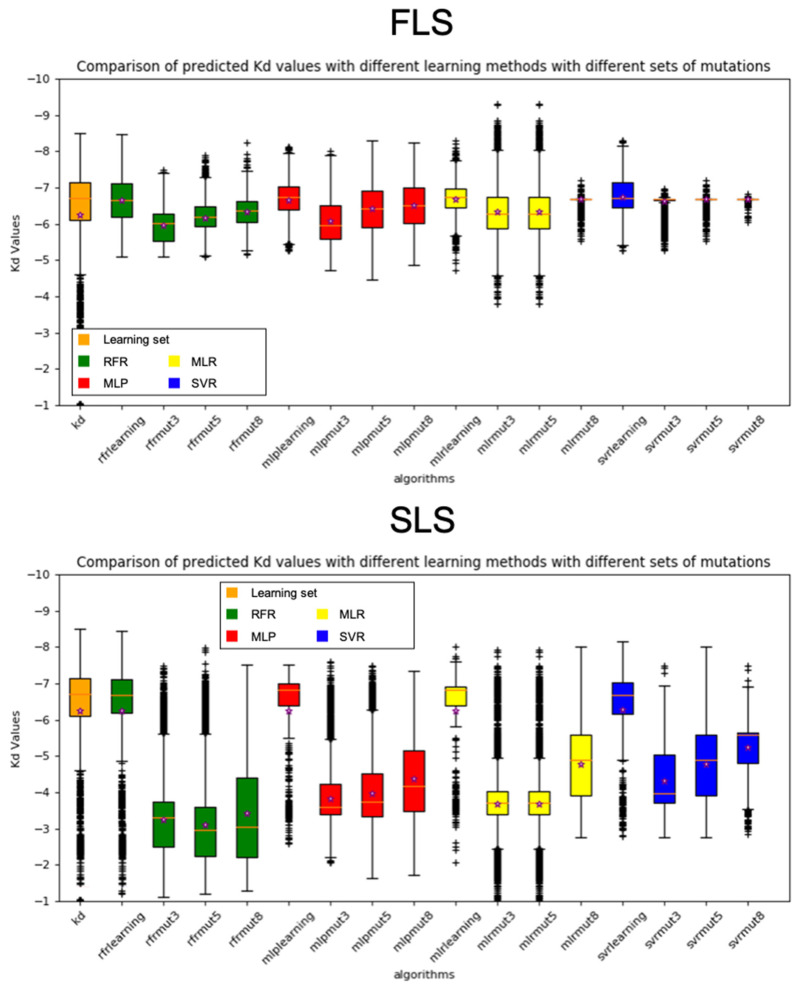
K_D_ distributions obtained with the four different algorithms in the two models for the three sets of random mutants. RFR in green, MLP in red, MLR in yellow, SVR in blue, trained either with FLS (**top**) or SLS (**bottom**) models. Purple star represents the average K_D_.

**Figure A1:**
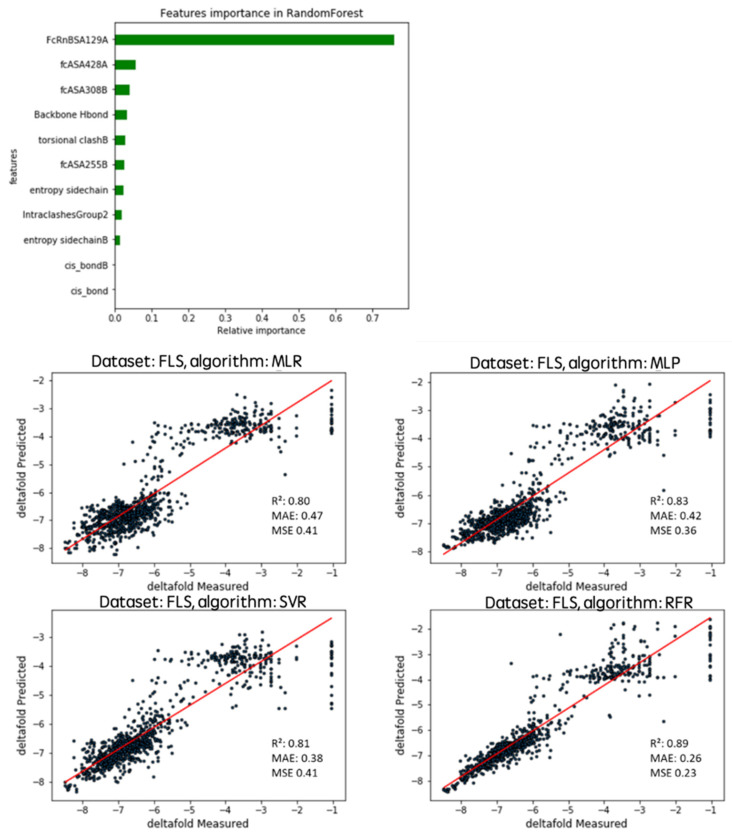
Model with FoldX.

To ensure that the models were not overfitting, despite good learning performance on the entire training datasets, we used a 10-fold cross-validation scheme. We performed this cross-validation test several times for each algorithm to ensure that scores were consistent between different runs, because each run of the algorithms can produce different results. With optimized parameters (see Materials and Methods), the consistent regression scores of the M1048/11 model (R^2^) obtained with MLR, MLP, SVR, and RFR are on average 0.45, 0.60, 0.75, and 0.84, respectively, and 0.77, 0.80, 0.82, and 0.88, respectively, with the M1251/6 model (Figure 2). The scores of MAE (mean absolute error) and MSE (mean squared error) are also ranked according to the best regression score, with the best scores obtained for the RFR. Although regression scores are better with the SLS model due to the larger range of K_D_ values in the training set, MAE and MSE increased significantly for all algorithms compared to the FLS model. We also shuffled K_D_ values in order to control the fit of our models. As expected, the correlation dropped drastically with R^2^ below 0 (R^2^ with no intercept can result in a negative value) while MAE and MSE increased dramatically at the same time for all algorithms. We also tested a model that also incorporated the energy terms (60 parameters) calculated from the FoldX suite v5.0 [29] with all variants and following the same procedure of removing duplicates and correlated features, but the performance did not improve, and the best correlation obtained was 0.89 with 11 parameters with the RFR (Figure A1).

### 2.3. Randomly Generated Variants Predicted Affinity Comparison with the Four Algorithms

For evaluating the capacity of our two models and algorithms to generalize to new data, we tested both models with the four algorithms with in silico randomly generated Fc variants. We generated two sets of more than 8000 variants containing three (mut3 set) and five (mut5 set) random mutations. These mutations were introduced at positions 251, 252, 253, 254, 255, 256, 257, 285, 286, 288, 307, 308, 309, 310, 311, 314, 428, 433, 434, 435, and 436 because the calculated features of our models only included these positions. We generated one additional set of 1000 Fc variants containing six to eight mutations (mut8 set), with not too much destabilizing, or with a positive effect on their own according to our dataset. The number of mutations was limited to eight because the effect of close mutations on the stability and production of the antibody is hard to predict.

We first compared the distribution of the predicted log K_D_ values for the SLS (1323 variants, σ 1.47, log K_D_ values range: [−1.03, −8.49] at pH 7.0) by the four algorithms (Figure 2). With the FLS model (Figure 2 top), the four algorithms have the same overall distributions but fail to reproduce the same distribution of log K_D_ values as the SLS set, in contrast to the SLS models (Figure 2 bottom).

Our two models did not reproduce the same distribution of log K_D_ values with the three sets of random mutants. With our two models, all the algorithms predicted that random variants of the mut3 and mut5 sets, but also variants of the mut8 set, would have on average less affinity at pH 7.0 than variants of the DS with a tendency to predict higher affinity for the mut8 set. The standard deviations and calculated log K_D_ means are far higher for the SLS model than for the M1048/11 model.

Interestingly, different algorithms yield different distributions of log K_D_, especially for the set of random variants (Figure 2). The RFR has the lowest log K_D_ mean predictions with the SLS and is the only algorithm that does not predict higher log K_D_ mean for the mut8 set. The MLP predicted the same type of distribution of K_D_ values as RFR with a tendency to predict higher values. The MLR is the algorithm with the highest standard deviation with the two models. Finally, the SVR showed a much narrower range of values with a standard deviation decreasing with the number of mutations with the first model in contrast to the second model.

### 2.4. Experimental Validation

**Figure A2:**
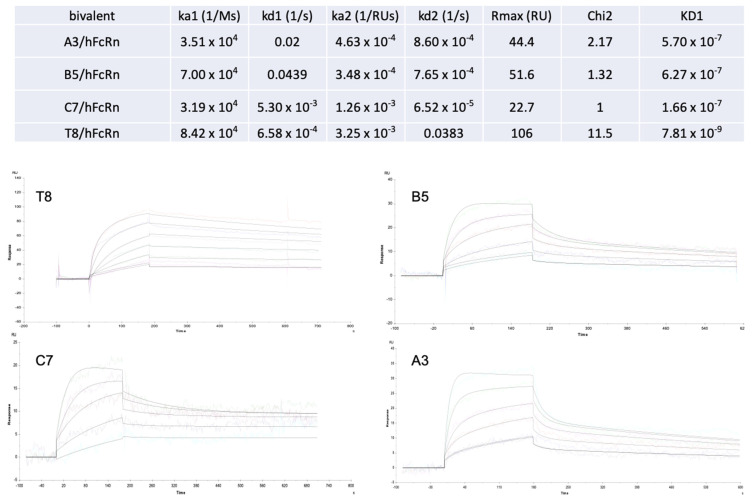
SPR experiments’ bivalent fit for variants A3, B5, C7, and T8.

**Figure A3:**
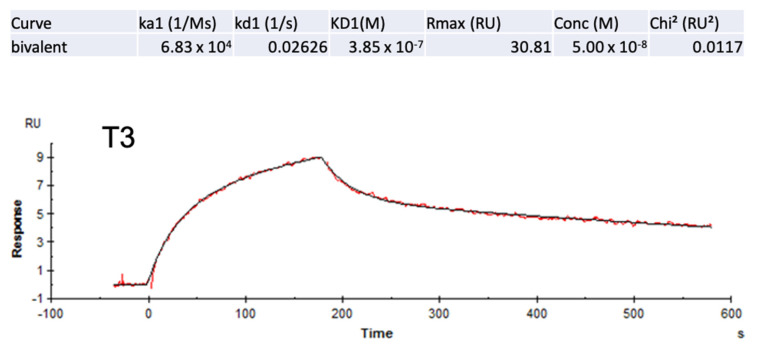
SPR measurements of variant T3 at pH 7.0 (bivalent fit).

To further validate our prediction method, we predicted the affinity of three new variants, which to our knowledge have never been tested. We then produced them and measured their affinities. We chose variants within our sets of in silico randomly generated variants (A3 (M252W/M428K/N434W), B5 (T256Y/H285Q/N286D/V308A/N434Y), C7 (T256E/N286H/K288E/V308P/L309D/N434Y/Y436K)) and introduced them in tocilizumab. For the control, we also generated two tocilizumab variants reported in the patent application: T8 (M252Y/N286E/T307Q/V308P/Q311A/N434Y/Y436V) and T3 (M252Y/T307D/N434Y). Our first two variants contain at least one substitution reported as a single destabilizing mutation in patents: the M428K for the mut3 variant and T256Y for the mut5 variant. The variant with seven mutations is a variant with a high predicted affinity by all the algorithms from the set of eight mutations. Affinities of the variants T8 and T3 measured in our SPR assay are close to the affinities reported in the patent application (Figure A2 and Figure A3). Overall, with the FLS and SLS, the four algorithms predict the affinity within a good range and are in good correlation with the measured affinities (Table 2, Figure A2 and Figure A3). In accordance with the 10-fold cross-validation results, the model poorly performs on the WT (tocilizumab) because it belongs to a class of antibodies with very weak binding for FcRn at neutral pH, whereas our model has better predictive potency for antibodies with affinities ranging from 1 × 10^−9^ to 1 × 10^−6^ for FcRn at neutral pH. The correlation of the six predicted vs actual measured affinities is better with the SLS model for the RFR, SVR, and MLR algorithms, in contrast to the MLP. However, the MAE is reduced for all algorithms (Table 3). For the new variants we produced, the SLS model has better performance than the FLS for all algorithms, especially for the SVR (Table 3). Overall, with the SLS model, the SVR algorithm has the best performance followed by the RFR, MLR, and MLP.

## 3. Discussion

Altogether, the present results show that it is possible to computationally predict the affinity for FcRn of Fc variants mutated at the interface of the Fc/FcRn complex with reasonable precision (+/−1 log). To do so, we carefully collected as many as possible publicly available Fc variants/FcRn affinity data by scrutinizing the scientific literature and relevant patents. Since differences exist between protocols used to measure the affinities, we built two different datasets. The smallest one includes only values obtained using a single protocol; the largest includes all available values. To build the two models based on these data, a large number of features relevant to the affinity prediction of a protein complex as well as features relevant for this particular type of complex were included. We also minimized as much as possible the overfitting by eliminating features that were too correlated between them in each learning set. To further optimize our procedure, we tested four algorithms. The results of these tests showed that random forest has the best capacity to adapt to our learning sets as compared to MLP, MLR, or SVR algorithms (with our hyper-parameters). Indeed, regression, MAE, and MSE scores are always better with this algorithm, regardless of the model used. This study also shows that the learning set has a high impact on the importance of features and on average predictions.

Not only are the models important but also the algorithms, as they show some variability in the predicted values and their distributions. It is, however, difficult to explain the variability between algorithms since their parameters are different. For example, the larger standard deviation of the MLR algorithm is probably due to its mathematical function, which is less sensitive to threshold effects than are MLP, SVR, and RFR. The MLP algorithm has been tuned with the tanh function (sigmoid function) and with an alpha parameter of 20 to limit overfitting. An alpha parameter of 0.1 would yield a larger range of value, but it would have a tendency to overfit the data. Algorithms with this kind of threshold are more relevant from a biochemical point of view, since the affinity of Fc variants is usually limited to 1 × 10^−10^, especially for random variants. This is important to keep in mind because if two algorithms are compared and have more or less the same performance in a cross-validation scheme, then it becomes difficult to decide which of them will better generalize to new data. It is also possible that an algorithm with good performance overfits to data, even with a cross-validation test, and will consequently have less capacity to generalize to new data than an algorithm with lesser performance on the same cross-validation test. For example, the RFR has the best performance in the cross-validation test, but the SVR has better performance with new variants. Moreover, the MLR has the worst performance on the cross-validation test, but it performs slightly better at predicting affinities for new variants than the MLP.

### 3.1. Model FLS

Our entire dataset is composed of 1323 variants. However, we built our FLS model selecting only homogenous data, derived from an accurate technique (SPR), in order to limit noise that could be induced by outliers. The drawback is that the FLS model is biased towards a particular type of variants, namely variants engineered to have better affinity at pH 7.0. Indeed, despite our efforts to get a maximum of unique variants from the patent database, our approach is still limited by the number and quality of data. For example, the exact K_D_ value of a variant described as a non-binder cannot be known, yet the impact of its mutations would certainly increase performance. In addition, companies tend to only publish good results, i.e., variants with better affinity, and not those with decreased affinity. This results in a dataset with a majority of variants with high affinities for FcRn, which decreases the performance in estimating low affinities. The quality and consistency of data is also a prerequisite of any model. However, the accuracy of measures may be low, especially for variants that are discarded from the first round of selection. Moreover, there are also sometimes discrepancies between studies reporting affinities. For example, in a recent study [17], mutation N434S has been reported to reduce the binding affinity of Fc to FcRn, whereas in patent US20100204454 this sole mutation has been reported to enhance the binding by threefold. Another effect of the dataset bias is that not only K_D_ but also the weight of the features could be over- or underestimated. The difference in importance of the features in this model can be explained by the composition of the FLS. Indeed, most of the variants of this learning set contain a hydrophobic amino acid at position 434, but they do not systematically have mutations in the region of the Fc near the β2m, which changes the number of interactions between the two molecules. As a result, this feature has a higher importance than the buried surface of residue 434 of the Fc. The relative importance of features with this model is also due to the absence of variants containing mutations at positions that are deeply buried (252, 253, and 310), explaining the very low importance (although crucial for the binding of the complex) of these positions in this model. 

### 3.2. Model SLS

It has been shown that antibodies binding to FcRn with affinities lower than 860 nM at physiologic pH have reduced half-lives [30]. Having data on the same variants at both acidic and physiological pH could help to better quantify the impact of this parameter. However, affinities at physiological pH are almost always reported as “no binding” because of the low sensitivity of the methods. It has been proposed that the pH impact was fairly linear between pH 6.0 and 7.4 on a log scale [24]; hence, a constant value could suffice to approximate the pH change. We made the second model M1251/6 with K_D_ at pH 7.0 based on this assumption, since all new examples of this second model were only reported at pH 6.0, or with no binding measure at neutral pH, and were mainly variants with a single destabilization mutation introducing an interpretation bias for the pH parameter (the algorithms interpret the diminution of pH as a factor reducing the binding). We homogenized the data by lowering the K_D_ of the examples reported only at pH 6.0 by 68-fold, since tocilizumab was reported to have a K_D_ of 1.3 × 10^−6^ M at pH 6.0 and 8.8 × 10^−5^ M at pH 7.0. Although this is a crude approximation, the correlation increased for all algorithms. However, the MAE and MSE increased, probably because the 68-fold change in K_D_ cannot be applied to all variants, or because these new examples had their affinities measured by less sensitive techniques such as ELISA. Indeed, we also evaluated the prediction of the four algorithms with our two models. The same transformation was applied on the reported affinities at pH 6.0, but the resulting precision for the described affinity was only +/−1.5 log K_D_ by the four algorithms with the second model (Table A2). In addition, if several histidine mutations are considered, the K_D_ change between the two pH values could be more drastic. In model M1251/6, the buried surface area of the FcRn amino acid 129 is the most discriminant feature (importance: 0.7) because most variants with no hydrophobic mutation at this position have decreased affinities for FcRn in the SLS. The weights of other features calculated by MLR, MLP, and SVR are negligible, which explains why the correlation curves of the second model show very little change in predicted K_D_s for large, measured K_D_ ranges and can cluster into two groups.

Cross-validation is the classical test to evaluate if a model does not overfit. Even if the algorithms performed well with the two models, both models are biased towards variants engineered to have high affinity at neutral pH as explained above. To evaluate the impact of this bias, we tested whether the models would reproduce the same distribution of predicted K_D_ of the learning set with the random variant sets (mut3 and mut5 sets). All the algorithms predicted ranges of values of lower affinity for the random variant sets than the learning set of the M1251/6 model (Figure 2). Conversely, the M1048/11 model tends to stick to the range of value of the learning set except for the SVR (Figure 2).

In contrast to the SVR and MLR, the RFR and MLP algorithms did not predict higher affinities within the set of eight “good” random mutations in which only individual mutations shown to increase the affinity were kept. However, some mutation combinations incorporated in this set might have decreased affinities.

We also compared the first 20 variants for each set with the higher predicted affinity, considering each algorithm. Most of the experimental Fc variants with significantly better affinity for FcRn at neutral pH have hydrophobic substitution at position 434, whereas histidine 310 and isoleucine 253 are not substituted. However, none of the algorithms tested shows this pattern in its top 20 ranked variants (Table A3).

We challenged our models with mutation combinations not diverging too much from the examples of the learning set. We choose two variants from the set of three and five mutations, each containing a destabilizing mutation. To ensure that we would be able to measure an affinity for these variants, they also had to contain at least one mutation which showed great improvement in affinity (such as the N434Y or N434W mutations) to counterbalance the negative effect on affinity. Although the chosen mutants do not diverge too much from the learning sets, the results of the experimental measurements show that we are able to accurately predict their affinities.

### 3.3. Further Improvements

Although our experimental validations show the reliability of the method, the robustness and predictive power of the models would be significantly increased with a larger experimental validation set. In addition, our DS comprises 1323 variants, but this number could be larger if we had taken into account intramolecular interaction or long-range effects. Indeed, some mutations that are not at the interaction surface can impact the affinity of the complex. For example, Booth et al. [16] hypothesized that M428L and A378V could stabilize the 250 pseudo-helix. They also proposed in their study to complement the positively charged N-terminal region of the FcRn β-domain with T256, T307, H285, N286, and N315. Other general descriptors to consider could be the electrostatic complementarity between regions of the complex or the rigidity of the 250 pseudo-helix. It has also been shown that the destabilization of the region of the Fc at low pH could be responsible for higher binding [31]. Although the reasons are not very well understood, Monnet et al. [15] showed that the positions that are not in the interaction site (264 and 389) could favorably impact the binding. More intriguingly, they have also shown that mutations far away from the interaction site (P230S, P228L, or P228R) could enhance FcRn binding, although not consistently. In the same way, Ternant et al. [5] reported the influence of four different G1m allotypes regarding FcRn binding, although amino acids 214, 356, and 358 are distant from the interaction site. Some of these mutations outside of the Fc/FcRn interaction site have been introduced for optimizing binding to Fcγ receptors (or already exist in natural sequences), and they could still have an impact on FcRn binding. These new parameters could thus enhance the performance of our method. 

As explained at the beginning of this paper, we chose using rather simple methods for learning because we did not know whether we had enough data, because we wanted to avoid overfitting, and because we wanted to demonstrate the validity of the global approach. The results bring positive answers to these three points, and it would now be worth trying more complex methods such as evolutionary algorithms or neural networks.

Finally, we focused on predicting the overall affinity (K_D_) because there were too few data on k_on_ and k_off_. However, to obtain variants with desirable properties, k_on_ and k_off_ should also be taken into account [24]. Indeed, it has been shown that the endosomal trafficking time of the antibody was very short (a half-life time less than 10 min). Thus, it would be important for an antibody to have a very high k_on_ at pH 6.0 rather than a low k_off_, which could prevent the antibody from being released back into the circulation. However, generated variants with a slow off-rate exhibited an extended half-life in mice and cynomolgus monkeys [16]. In any case, integrating these data could help to improve in silico design methods.

## 4. Materials and Methods

### 4.1. Antibody Expression and Purification

T3, T8, A3, B5, and C7 antibodies were produced by RD-Biotech (Besançon, France) following standard procedures by transient transfection of CHO cells. Antibodies were purified with protein A.

### 4.2. Surface Plasmon Resonance

SPR experiments were performed on Bia3000 apparatus at 25 °C in 50 mM phosphate buffer with 150 mM NaCl containing 0.05% P20 surfactant (GE Healthcare, Chicago, IL, USA) adjusted at pH 7 or pH 6 as required. hFcRn (Immunitrack, Copenhagen, Denmark) was immobilized in acetate buffer at pH 5 on CM5 sensor chips at a level lower than 200 RU. Increasing concentrations of antibody variants were injected over 180 s. After a dissociation phase of 400 s, the FcRn-coated sensor chip was regenerated by a pulse of 10 mM NaOH and PBS. The multi-cycle kinetics were evaluated by a bivalent model fitting (BiaEvaluation 4.1.1, GE Healthcare). Each variant was analyzed on freshly immobilized hFcRn.

### 4.3. Structure-Based Feature Extraction

To model the 3D structures of the Fc mutants, the 4N0U.pdb file was used as a template. Using the mutagenesis tool from PyMOL v2.5.4, the 3D structure of the complex between FcRn and each mutant from the dataset was generated and exported as a pdb file. CCP4 software v8.0.009 was used to compute the different features used in the algorithms. Features calculated for each residue by CCP4 were: BSA (buried surface area), ASA (accessible surface area), and solvation energy. General features calculated by CCP4 for the whole complex were: number of interface residues, ∆G (solvation energy gain score), p-value (hydrophobic score), BE (theoretical binding energy), and number of hydrogen and salt bridges between interfaces. Total number of hydrogen bonds (cutoff: 3.5 angstroms), total number of salt bridges (cutoff: 4.0 angstroms), total number of contacts between amino acids’ cα (cutoff: 4.0 angstroms), average distance between hydrogen bonds and number of paired hydrophilic amino acids were also added in addition to CCP4-calculated parameters.

Algorithms from scikit-learn v0.20.3 were used. Data were standardized.

The estimator’s parameters were set to: 

RFR: (n_estimators = ‘warn’, criterion = ‘mse’, max_depth = 10, min_samples_split = 2, min_samples_leaf = 1, min_weight_fraction_leaf = 0.0, max_features = ‘auto’, max_leaf_nodes = None, min_impurity_decrease = 0.0, min_impurity_split = None, bootstrap = True, oob_score = False, n_jobs = None, random_state = None, verbose = 0, warm_start = False).

SVR: (kernel = ‘rbf’, degree = 3, gamma = ‘auto_deprecated’, coef0 = 0.0, tol = 0.001, C = 1.0, epsilon = 0.1, shrinking = True, cache_size = 200, verbose = False, max_iter = −1).

MLPRegressor: (solver = ‘lbfgs’, alpha = 20, hidden_layer_sizes = (20,2), random_state = 10, activation = ‘tanh’, max_iter = 4000, tol = 0.00001, early_stopping = True).

LR: (fit_intercept = True, normalize = False, copy_X = True, n_jobs = None).

## 5. Conclusions

Affinity prediction is one of the toughest bioinformatics challenges, and although progress has been made, there is still room for improvement. We chose to focus on one particular protein complex type for which many data were available. The results of the training show that this kind of approach is appropriate and also that the diversity of the training set is crucial to avoid bias and to correctly evaluate the importance of the different features. Despite all the limitations of our models, we were able to correctly predict the affinities of the three variants that were produced in this study. However, the obtained results do not allow us to make an educated choice between the methods. The SLS-trained algorithms appear to perform better than the FLS-trained ones, both in 10-fold cross-validation (Figure 1) and in predicting the affinities of the new variants (Table 2 and Table 3). However, the MLS and MLP algorithms perform better in predicting the new variants, but the RFR algorithm is better in the 10-fold cross-validation. Thus, deciding between the three methods will require more validations.

The advantage of this method is that it does not require initial knowledge to generate in silico random variants and select mutants with high affinity. However, like most artificial-intelligence-based methods, it does not explain how various combinations of mutations can modulate the affinity of the Fc to FcRn. Still, it provides new interesting combinations of mutations while reducing the number of variants to test.

## Figures and Tables

**Table 1:** Datasets and machine learning methods used.

**Datasets**		
**Name**	**Number of Variants**	**Selection Criteria**
First learning set (FLS)	1099	Affinities measured by SPR at 25 °C, pH 7.
Second learning set (SLS)	1323	FLS variants + 224 variants with affinities only measured at pH 6.
**Algorithms**		
**Name**	**Description**	
Support vector regressor (SVR)	The objective of support vector machines (SVMs) is to find the hyperplane separating at best the two categories of instances defined in a training sample. Support vector regression (SVR) uses the same principle, adding a constraint on the maximal distance between the instances and the hyperplane.	
Multi-linear regression (MLR)	Multiple linear regression optimizes a linear function of the parameters.	
Multi-layer perceptron (MLP)	An MLP is a class of feedforward artificial neural networks (ANNs) with at least three layers of nodes (input, hidden, and output) and the neurons of hidden and output layers using non-linear activation functions.	
Random forest regressor (RFR)	A random forest is a meta-estimator that fits a number of classifying decision trees on various sub-samples of the dataset and uses averaging to improve the predictive accuracy and control overfitting.	

**Table 2:** Comparison of predicted versus experimental affinities at pH 7.0 for 3 randomly generated variants. The 3 variants have 3, 5, and 7 mutations and are predicted with the two different models and with 4 different algorithms. Measured affinities at pH 6.0 are also shown. Cells in green, yellow, and red correspond to very good (log err = |log(pred) − log(K_D_)| ≤ 0.1), correct (0.1 < log err ≤ 1), and incorrect (log err > 1) predictions, respectively. Statistical analysis is given in Table 3.

	Tocilizumab *	T8 *	T3 *	C7	B5	A3
Mutations	None	M252Y/N286E/T307Q/V308P/Q311A/N434Y/Y436V	M252Y/T307D/N434Y	T256E/N286H/K288E/V308P/L309D/N434Y/Y436K	T256Y/H285Q/N286D/V308A/N434Y	M252W/M428K/N434W
K_D_ at pH7 (patent)	8.8 × 10^−5^	4.4 × 10^−9^	2.1 × 10^−7^			
K_D_ at pH7 (this work)	NB	7.8 × 10^−9^	3.8 × 10^−7^	1.6 × 10^−7^	6.2 × 10^−7^	5.7 × 10^−7^
K_D_ at pH6 (this work)	3.8 × 10^−7^	1.3 × 10^−9^	1.3 × 10^−8^	3.4 × 10^−8^	4.5 × 10^−8^	1.1 × 10^−8^
**Prediction setting**						
SVR/FLS	6.91 × 10^−7^	7.29 × 10^−9^	2.54 × 10^−8^	1.90 × 10^−7^	1.90 × 10^−7^	1.40 × 10^−7^
SVR/SLS	6.70 × 10^−6^	1.30 × 10^−8^	9.50 × 10^−8^	2.30 × 10^−7^	4.20 × 10^−7^	4.20 × 10^−7^
MLR/FLD	6.20 × 10^−7^	1.30 × 10^−8^	8.00 × 10^−8^	1.80 × 10^−8^	4.40 × 10^−8^	1.30 × 10^−7^
MLR/SLS	1.00 × 10^−4^	5.40 × 10^−8^	6.50 × 10^−8^	7.40 × 10^−8^	1.40 × 10^−7^	2.70 × 10^−7^
MLP/FLS	8.00 × 10^−7^	1.30 × 10^−8^	8.00 × 10^−8^	8.70 × 10^−8^	1.30 × 10^−7^	7.70 × 10^−8^
MLP/SLS	1.90 × 10^−5^	4.40 × 10^−8^	6.00 × 10^−8^	6.80 × 10^−7^	7.10 × 10^−6^	6.90 × 10^−7^
RFR/FLS	1.20 × 10^−6^	3.80 × 10^−9^	2.47 × 10^−7^	6.00 × 10^−8^	1.60 × 10^−7^	3.30 × 10^−7^
RFR/SLS	3.40 × 10^−6^	4.10 × 10^−9^	1.50 × 10^−7^	4.90 × 10^−8^	2.10 × 10^−7^	3.20 × 10^−7^

* Tocilizumab, T8, and T3 were removed from the learning set in each prediction setting.

**Table 3:** Comparison between MAE, Pearson correlation coefficient, and maximum error between predictions at pH 7.0 and measurements for the 6 antibodies of Table 2 or only for the 3 produced variants (Mut3, Mut5, and Mut8).

	SVR/FLS	SVR/SLS	MLR/FLS	MLR/SLS	MLP/FLS	MLP/SLS	RFR/FLS	RFR/SLS
Log K_D_ MAE (6 Abs)	0.64	0.19	0.81	0.11	0.63	0.26	0.52	0.47
Pearson correlation coefficient (6 Abs)	0.88	0.98	0.91	0.89	0.98	0.84	0.91	0.97
Log K_D_ Maximum error (6 Abs)	2.11	1.12	2.15	1.09	2.04	1.06	1.87	1.41
Log K_D_ MAE (A, B, C Abs)	0.35	0.05	0.91	0.44	0.60	0.59	0.42	0.41
Pearson correlation coefficient (A, B, C Abs)	−0.45	0.99	0.81	0.83	0.35	0.55	0.88	0.96
Log K_D_ Maximum error (A, B, C Abs)	0.61	0.17	1.15	0.65	0.87	1.06	0.59	0.51

## Data Availability

Not applicable.

## Appendix A

**Table A1:** List of descriptors investigated.

No.	Feature Name	Meaning
1	FcSOLV251A	Solvation effect of the 251 residue in Å^2^
2	FcSOLV252A	Solvation effect of the 252 residue in Å^2^
3	FcSOLV253A	Solvation effect of the 253 residue in Å^2^
4	FcSOLV254A	Solvation effect of the 254 residue in Å^2^
5	FcSOLV309A	Solvation effect of the 309 residue in Å^2^
6	FcSOLV310A	Solvation effect of the 310 residue in Å^2^
7	FcSOLV311A	Solvation effect of the 311 residue in Å^2^
8	FcSOLV314A	Solvation effect of the 314 residue in Å^2^
9	FcSOLV428A	Solvation effect of the 428 residue in Å^2^
10	FcSOLV433A	Solvation effect of the 433 residue in Å^2^
11	FcSOLV434A	Solvation effect of the 434 residue in Å^2^
12	FcSOLV435A	Solvation effect of the 435 residue in Å^2^
13	FcSOLV436A	Solvation effect of the 436 residue in Å^2^
14	FcSOLV253B	Solvation effect of the 253 residue in Å^2^
15	FcSOLV255B	Solvation effect of the 255 residue in Å^2^
16	FcSOLV256B	Solvation effect of the 256 residue in Å^2^
17	FcSOLV257B	Solvation effect of the 257 residue in Å^2^
18	FcSOLV285B	Solvation effect of the 285 residue in Å^2^
19	FcSOLV286B	Solvation effect of the 286 residue in Å^2^
20	FcSOLV288B	Solvation effect of the 288 residue in Å^2^
21	FcSOLV307B	Solvation effect of the 307 residue in Å^2^
22	FcSOLV308	Solvation effect of the 308 residue in Å^2^
23	FcSOLV309B	Solvation effect of the 309 residue in Å^2^
24	FcSOLV310B	Solvation effect of the 310 residue in Å^2^
25	FcBSA251A	Buried surface of the 251 residue in Å^2^
26	FcBSA252A	Buried surface of the 252 residue in Å^2^
27	FcBSA253A	Buried surface of the 253 residue in Å^2^
28	FcBSA254A	Buried surface of the 254 residue in Å^2^
29	FcBSA309A	Buried surface of the 309 residue in Å^2^
30	FcBSA310A	Buried surface of the 310 residue in Å^2^
31	FcBSA311A	Buried surface of the 311 residue in Å^2^
32	FcBSA314A	Buried surface of the 314 residue in Å^2^
33	FcBSA428A	Buried surface of the 428 residue in Å^2^
34	FcBSA433A	Buried surface of the 433 residue in Å^2^
35	FcBSA434A	Buried surface of the 434 residue in Å^2^
36	FcBSA435A	Buried surface of the 435 residue in Å^2^
37	FcBSA436A	Buried surface of the 436 residue in Å^2^
38	FcBSA253B	Buried surface of the 253 residue in Å^2^
39	FcBSA255B	Buried surface of the 255 residue in Å^2^
40	FcBSA256B	Buried surface of the 256 residue in Å^2^
41	FcBSA257B	Buried surface of the 257 residue in Å^2^
42	FcBSA285B	Buried surface of the 285 residue in Å^2^
43	FcBSA286B	Buried surface of the 286 residue in Å^2^
44	FcBSA288B	Buried surface of the 288 residue in Å^2^
45	FcBSA307B	Buried surface of the 307 residue in Å^2^
46	FcBSA308	Buried surface of the 308 residue in Å^2^
47	FcBSA309B	Buried surface of the 309 residue in Å^2^
48	FcBSA310B	Buried surface of the 310 residue in Å^2^
49	FcASA251A	Surface accessible to the solvent of the 251 residue in Å^2^
50	FcASA252A	Surface accessible to the solvent of the 252 residue in Å^2^
51	FcASA253A	Surface accessible to the solvent of the 253 residue in Å^2^
52	FcASA254A	Surface accessible to the solvent of the 254 residue in Å^2^
53	FcASA309A	Surface accessible to the solvent of the 309 residue in Å^2^
54	FcASA310A	Surface accessible to the solvent of the 310 residue in Å^2^
55	FcASA311A	Surface accessible to the solvent of the 311 residue in Å^2^
56	FcASA314A	Surface accessible to the solvent of the 314 residue in Å^2^
57	FcASA428A	Surface accessible to the solvent of the 428 residue in Å^2^
58	FcASA433A	Surface accessible to the solvent of the 433 residue in Å^2^
59	FcASA434A	Surface accessible to the solvent of the 434 residue in Å^2^
60	FcASA435A	Surface accessible to the solvent of the 435 residue in Å^2^
61	FcASA436A	Surface accessible to the solvent of the 436 residue in Å^2^
62	FcASA253B	Surface accessible to the solvent of the 253 residue in Å^2^
63	FcASA255B	Surface accessible to the solvent of the 255 residue in Å^2^
64	FcASA256B	Surface accessible to the solvent of the 256 residue in Å^2^
65	FcASA257B	Surface accessible to the solvent of the 257 residue in Å^2^
66	FcASA285B	Surface accessible to the solvent of the 285 residue in Å^2^
67	FcASA286B	Surface accessible to the solvent of the 286 residue in Å^2^
68	FcASA288B	Surface accessible to the solvent of the 288 residue in Å^2^
69	FcASA307B	Surface accessible to the solvent of the 307 residue in Å^2^
70	FcASA308B	Surface accessible to the solvent of the 308 residue in Å^2^
71	FcASA309B	Surface accessible to the solvent of the 309 residue in Å^2^
72	FcASA310B	Surface accessible to the solvent of the 310 residue in Å^2^
73	FcRnSOLV88A	Solvation effect of the 88
74	FcRnSOLV112A	Solvation effect of the 112
75	FcRnSOLV113A	Solvation effect of the 113
76	FcRnSOLV114A	Solvation effect of the 114
77	FcRnSOLV115A	Solvation effect of the 115
78	FcRnSOLV116A	Solvation effect of the 116
79	FcRnSOLV128A	Solvation effect of the 128
80	FcRnSOLV129A	Solvation effect of the 129
81	FcRnSOLV130A	Solvation effect of the 130
82	FcRnSOLV131A	Solvation effect of the 131
83	FcRnSOLV132A	Solvation effect of the 132
84	FcRnSOLV133A	Solvation effect of the 133
85	FcRnSOLV135A	Solvation effect of the 135
86	FcRnSOLV1B	Solvation effect of the 1
87	FcRnSOLV2B	Solvation effect of the 2
88	FcRnSOLV3B	Solvation effect of the 3
89	FcRnSOLV4B	Solvation effect of the 4
90	FcRnSOLV85B	Solvation effect of the 85
91	FcRnSOLV86B	Solvation effect of the 86
92	FcRnBSA88A	Buried surface of the 88
93	FcRnBSA112A	Buried surface of the 112
94	FcRnBSA113A	Buried surface of the 113
95	FcRnBSA114A	Buried surface of the 114
96	FcRnBSA115A	Buried surface of the 115
97	FcRnBSA116A	Buried surface of the 116
98	FcRnBSA128A	Buried surface of the 128
99	FcRnBSA129A	Buried surface of the 129
100	FcRnBSA130A	Buried surface of the 130
101	FcRnBSA131A	Buried surface of the 131
102	FcRnBSA132A	Buried surface of the 132
103	FcRnBSA133A	Buried surface of the 133
104	FcRnBSA135A	Buried surface of the 135
105	FcRnBSA1B	Buried surface of the 1
106	FcRnBSA2B	Buried surface of the 2
107	FcRnBSA3B	Buried surface of the 3
108	FcRnBSA4B	Buried surface of the 4
109	FcRnBSA85B	Buried surface of the 85
110	FcRnBSA86B	Buried surface of the 86
111	FcRnASA88A	Surface accessible to the solvent of the 88
112	FcRnASA112A	Surface accessible to the solvent of the 112
113	FcRnASA113A	Surface accessible to the solvent of the 113
114	FcRnASA114A	Surface accessible to the solvent of the 114
115	FcRnASA115A	Surface accessible to the solvent of the 115
116	FcRnASA116A	Surface accessible to the solvent of the 116
117	FcRnASA128A	Surface accessible to the solvent of the 128
118	FcRnASA129A	Surface accessible to the solvent of the 129
119	FcRnASA130A	Surface accessible to the solvent of the 130
120	FcRnASA131A	Surface accessible to the solvent of the 131
121	FcRnASA132A	Surface accessible to the solvent of the 132
122	FcRnASA133A	Surface accessible to the solvent of the 133
123	FcRnASA135A	Surface accessible to the solvent of the 135
124	FcRnASA1B	Surface accessible to the solvent of the 1
125	FcRnASA2B	Surface accessible to the solvent of the 2
126	FcRnASA3B	Surface accessible to the solvent of the 3
127	FcRnASA4B	Surface accessible to the solvent of the 4
128	FcRnASA85B	Surface accessible to the solvent of the 85
129	FcRnASA86B	Surface accessible to the solvent of the 86
130	nbaainterFcA	Number of atoms interacting between Fc and the FcRns alpha chain
131	nbaainterFcB	Number of atoms interacting between Fc and the FcRns beta chain
132	nbliaiHFcA	Number of hydrogen bonds between Fc and the FcRns alpha chain
133	nbliaiHFcB	Number of hydrogen bonds between Fc and the FcRns beta chain
134	nbsaltFcA	Number of salt bridges between Fc and the FcRns alpha chain
135	nbsaltFcB	Number of salt bridges between Fc and the FcRns beta chain
136	interFace_solv_en_FcA	Solvation energy gain score calculated by PISA between Fc and the FcRns alpha chain
137	interface_solv_en_FcB	Solvation energy gain score calculated by PISA between Fc and the FcRns beta chain
138	p_valueFcA	Hydrophobic score calculated by PISA between Fc and the FcRns alpha chain
139	p_valueFcB	Hydrophobic score calculated by PISA between Fc and the FcRns beta chain
140	delta_g_theoriqueFcA	Theoretical binding energy score calculated by PISA between Fc and the FcRns alpha chain
141	delta_g_theoriqueFcB	Theoretical binding energy score calculated by PISA between Fc and the FcRns beta chain
142	Bond Strength	Average distance between bonds
143	paired hydrophilic	Number of paired hydrophilic amino acids
144	pH	pH
145	nbr_bounds_h	Total number of hydrogen bonds
146	nbr_ bounds _s	Total number of salt bridges
147	nbr_ bounds _c	Total number of contacts between amino acids’ cα atoms

A and B stand for FcRns α chain and β chain, respectively.

**Table A2:** Predictions of affinities at pH 7 of variants for which the measure has been done at pH 6.0 reported in [17].

Variant #	K_D_ pH 7 Predicted	K_D_ pH 7 Computed	K_D_ pH 6 Measured
**RFR**			
T256E/T307Q	4.06 × 10^−5^	1.58 × 10^−5^	2.32 × 10^−7^
T256D/T307W	1.78 × 10^−4^	1.15 × 10^−5^	1.69 × 10^−7^
M252Y/T256D	3.23 × 10^−5^	6.39 × 10^−6^	9.40 × 10^−8^
M252Y/T256E	1.87 × 10^−6^	8.70 × 10^−6^	1.28 × 10^−7^
M252Y/T307W	2.31 × 10^−5^	8.02 × 10^−6^	1.18 × 10^−7^
M252Y/T256D/T307Q	5.48 × 10^−7^	7.82 × 10^−6^	1.15 × 10^−7^
M252Y/T256E/T307Q	6.69 × 10^−7^	1.48 × 10^−5^	2.18 × 10^−7^
**MLP**			
T256E/T307Q	4.45 × 10^−5^	1.58 × 10^−5^	2.32 × 10^−7^
T256D/T307W	4.50 × 10^−5^	1.15 × 10^−5^	1.69 × 10^−7^
M252Y/T256D	8.66 × 10^−6^	6.39 × 10^−6^	9.40 × 10^−8^
M252Y/T256E	8.74 × 10^−6^	8.70 × 10^−6^	1.28 × 10^−7^
M252Y/T307W	1.33 × 10^−6^	8.02 × 10^−6^	1.18 × 10^−7^
M252Y/T256D/T307Q	1.46 × 10^−6^	7.82 × 10^−6^	1.15 × 10^−7^
M252Y/T256E/T307Q	1.48 × 10^−6^	1.48 × 10^−5^	2.18 × 10^−7^
**MLR**			
T256E/T307Q	9.25 × 10^−5^	1.58 × 10^−5^	2.32 × 10^−7^
T256D/T307W	1.03 × 10^−4^	1.15 × 10^−5^	1.69 × 10^−7^
M252Y/T256D	7.33 × 10^−5^	6.39 × 10^−6^	9.40 × 10^−8^
M252Y/T256E	6.92 × 10^−5^	8.70 × 10^−6^	1.28 × 10^−7^
M252Y/T307W	3.00 × 10^−5^	8.02 × 10^−6^	1.18 × 10^−7^
M252Y/T256D/T307Q	2.81 × 10^−5^	7.82 × 10^−6^	1.15 × 10^−7^
M252Y/T256E/T307Q	2.72 × 10^−5^	1.48 × 10^−5^	2.18 × 10^−7^
**SVR**			
T256E/T307Q	9.25 × 10^−5^	1.58 × 10^−5^	2.32 × 10^−7^
T256D/T307W	1.03 × 10^−4^	1.15 × 10^−5^	1.69 × 10^−7^
M252Y/T256D	7.33 × 10^−5^	6.39 × 10^−6^	9.40 × 10^−8^
M252Y/T256E	6.92 × 10^−5^	8.70 × 10^−6^	1.28 × 10^−7^
M252Y/T307W	3.00 × 10^−5^	8.02 × 10^−6^	1.18 × 10^−7^
M252Y/T256D/T307Q	2.81 × 10^−5^	7.82 × 10^−6^	1.15 × 10^−7^
M252Y/T256E/T307Q	2.72 × 10^−5^	1.48 × 10^−5^	2.18 × 10^−7^

**Table A3:** For the two different models and the four different algorithms, 20 variants predicted with the highest K_D_.

Model FL		
**RFR Learning_Set**		
**Variant #**	**Mutations**	**K_D_**
833	235R, 239K, 252Y, 286E, 307Q, 308P, 311A, 428I, 434Y, 436V	3.46 × 10^−9^
831	250V, 252Y, 286E, 307Q, 308P, 311A, 428I, 434Y, 436V	3.61 × 10^−9^
802	235R, 239K, 252Y, 286E, 307Q, 308P, 311A, 434Y, 436V	3.70 × 10^−9^
829	235K, 239K, 252Y, 286E, 307Q, 308P, 311A, 434Y, 436V	3.70 × 10^−9^
832	235R, 239K, 250V, 252Y, 286E, 307Q, 308P, 311A, 428I, 434Y, 436V	3.77 × 10^−9^
800	250V, 252Y, 286E, 307Q, 308P, 311A, 434Y, 436V	3.87 × 10^−9^
801	235R, 239K, 250V, 252Y, 286E, 307Q, 308P, 311A, 434Y, 436V	4.06 × 10^−9^
828	235K, 239K, 250V, 252Y, 286E, 307Q, 308P, 311A, 434Y, 436V	4.06 × 10^−9^
568	252Y, 286E, 307Q, 308P, 311A, 428I, 434Y	4.24 × 10^−9^
1	239K, 252Y, 270F, 286E, 307Q, 308P, 311A, 428I, 434Y	4.55 × 10^−9^
2	239K, 252W, 286E, 308P, 428Y, 434Y	6.50 × 10^−9^
3	239K, 252W, 256E, 286E, 308P, 428Y, 434Y	6.51 × 10^−9^
567	252Y, 286E, 307Q, 308P, 311A, 434Y	6.78 × 10^−9^
527	239K, 252Y, 286E, 307Q, 308P, 311A, 434Y	7.15 × 10^−9^
5	239K, 252Y, 270F, 286E, 307Q, 308P, 428I, 434Y	7.45 × 10^−9^
7	239K, 252Y, 270F, 286E, 308P, 387E, 428I, 434Y	7.47 × 10^−9^
8	239K, 252Y, 270F, 286E, 308P, 428I, 434Y	7.63 × 10^−9^
4	239K, 252Y, 270F, 286E, 308P, 311A, 428I, 434Y	7.68 × 10^−9^
565	252Y, 286E, 308P, 428I, 434Y	7.92 × 10^−9^
6	239K, 252Y, 286E, 308P, 428I, 434Y	7.97 × 10^−9^
**MLP learning_set**		
**Variant #**	**Mutations**	**K_D_**
568	235R, 239K, 252Y, 286E, 307Q, 308P, 311A, 428I, 434Y, 436V	7.65 × 10^−9^
1	250V, 252Y, 286E, 307Q, 308P, 311A, 428I, 434Y, 436V	7.65 × 10^−9^
29	235R, 239K, 252Y, 286E, 307Q, 308P, 311A, 434Y, 436V	7.98 × 10^−9^
5	235K, 239K, 252Y, 286E, 307Q, 308P, 311A, 434Y, 436V	8.07 × 10^−9^
802	235R, 239K, 250V, 252Y, 286E, 307Q, 308P, 311A, 428I, 434Y, 436V	8.19 × 10^−9^
829	250V, 252Y, 286E, 307Q, 308P, 311A, 434Y, 436V	8.19 × 10^−9^
800	235R, 239K, 250V, 252Y, 286E, 307Q, 308P, 311A, 434Y, 436V	8.32 × 10^−9^
801	235K, 239K, 250V, 252Y, 286E, 307Q, 308P, 311A, 434Y, 436V	8.32 × 10^−9^
828	252Y, 286E, 307Q, 308P, 311A, 428I, 434Y	8.32 × 10^−9^
831	239K, 252Y, 270F, 286E, 307Q, 308P, 311A, 428I, 434Y	8.66 × 10^−9^
832	239K, 252W, 286E, 308P, 428Y, 434Y	8.66 × 10^−9^
19	239K, 252W, 256E, 286E, 308P, 428Y, 434Y	8.69 × 10^−9^
47	252Y, 286E, 307Q, 308P, 311A, 434Y	8.72 × 10^−9^
24	239K, 252Y, 286E, 307Q, 308P, 311A, 434Y	8.72 × 10^−9^
495	239K, 252Y, 270F, 286E, 307Q, 308P, 428I, 434Y	8.72 × 10^−9^
833	239K, 252Y, 270F, 286E, 308P, 387E, 428I, 434Y	9.13 × 10^−9^
567	239K, 252Y, 270F, 286E, 308P, 428I, 434Y	9.44 × 10^−9^
527	239K, 252Y, 270F, 286E, 308P, 311A, 428I, 434Y	9.44 × 10^−9^
3	252Y, 286E, 308P, 428I, 434Y	1.02 × 10^−8^
570	239K, 252Y, 286E, 308P, 428I, 434Y	1.09 × 10^−8^
**MLR learning_set**		
**Variant #**	**Mutations**	**K_D_**
544	235R, 239K, 252Y, 286E, 307Q, 308P, 311A, 428I, 434Y, 436V	5.13 × 10^−9^
530	250V, 252Y, 286E, 307Q, 308P, 311A, 428I, 434Y, 436V	6.30 × 10^−9^
244	235R, 239K, 252Y, 286E, 307Q, 308P, 311A, 434Y, 436V	7.93 × 10^−9^
247	235K, 239K, 252Y, 286E, 307Q, 308P, 311A, 434Y, 436V	8.08 × 10^−9^
343	235R, 239K, 250V, 252Y, 286E, 307Q, 308P, 311A, 428I, 434Y, 436V	8.10 × 10^−9^
543	250V, 252Y, 286E, 307Q, 308P, 311A, 434Y, 436V	9.51 × 10^−9^
568	235R, 239K, 250V, 252Y, 286E, 307Q, 308P, 311A, 434Y, 436V	9.56 × 10^−9^
1	235K, 239K, 250V, 252Y, 286E, 307Q, 308P, 311A, 434Y, 436V	9.69 × 10^−9^
5	252Y, 286E, 307Q, 308P, 311A, 428I, 434Y	9.77 × 10^−9^
567	239K, 252Y, 270F, 286E, 307Q, 308P, 311A, 428I, 434Y	1.10 × 10^−8^
527	239K, 252W, 286E, 308P, 428Y, 434Y	1.11 × 10^−8^
29	239K, 252W, 256E, 286E, 308P, 428Y, 434Y	1.11 × 10^−8^
47	252Y, 286E, 307Q, 308P, 311A, 434Y	1.12 × 10^−8^
833	239K, 252Y, 286E, 307Q, 308P, 311A, 434Y	1.16 × 10^−8^
24	239K, 252Y, 270F, 286E, 307Q, 308P, 428I, 434Y	1.18 × 10^−8^
495	239K, 252Y, 270F, 286E, 308P, 387E, 428I, 434Y	1.18 × 10^−8^
19	239K, 252Y, 270F, 286E, 308P, 428I, 434Y	1.27 × 10^−8^
802	239K, 252Y, 270F, 286E, 308P, 311A, 428I, 434Y	1.31 × 10^−8^
829	252Y, 286E, 308P, 428I, 434Y	1.31 × 10^−8^
536	239K, 252Y, 286E, 308P, 428I, 434Y	1.45 × 10^−8^
**SVR learning_set**		
**Variant #**	**Mutations**	**K_D_**
831	235R, 239K, 252Y, 286E, 307Q, 308P, 311A, 428I, 434Y, 436V	4.90 × 10^−9^
832	250V, 252Y, 286E, 307Q, 308P, 311A, 428I, 434Y, 436V	4.90 × 10^−9^
800	235R, 239K, 252Y, 286E, 307Q, 308P, 311A, 434Y, 436V	5.24 × 10^−9^
801	235K, 239K, 252Y, 286E, 307Q, 308P, 311A, 434Y, 436V	5.24 × 10^−9^
828	235R, 239K, 250V, 252Y, 286E, 307Q, 308P, 311A, 428I, 434Y, 436V	5.24 × 10^−9^
833	250V, 252Y, 286E, 307Q, 308P, 311A, 434Y, 436V	5.87 × 10^−9^
2	235R, 239K, 250V, 252Y, 286E, 307Q, 308P, 311A, 434Y, 436V	6.92 × 10^−9^
3	235K, 239K, 250V, 252Y, 286E, 307Q, 308P, 311A, 434Y, 436V	7.08 × 10^−9^
802	252Y, 286E, 307Q, 308P, 311A, 428I, 434Y	7.28 × 10^−9^
829	239K, 252Y, 270F, 286E, 307Q, 308P, 311A, 428I, 434Y	7.28 × 10^−9^
5	239K, 252W, 286E, 308P, 428Y, 434Y	8.13 × 10^−9^
565	239K, 252W, 256E, 286E, 308P, 428Y, 434Y	8.71 × 10^−9^
6	252Y, 286E, 307Q, 308P, 311A, 434Y	8.71 × 10^−9^
7	239K, 252Y, 286E, 307Q, 308P, 311A, 434Y	8.71 × 10^−9^
8	239K, 252Y, 270F, 286E, 307Q, 308P, 428I, 434Y	8.71 × 10^−9^
763	239K, 252Y, 270F, 286E, 308P, 387E, 428I, 434Y	1.02 × 10^−8^
818	239K, 252Y, 270F, 286E, 308P, 428I, 434Y	1.02 × 10^−8^
783	239K, 252Y, 270F, 286E, 308P, 311A, 428I, 434Y	1.02 × 10^−8^
567	252Y, 286E, 308P, 428I, 434Y	1.20 × 10^−8^
527	239K, 252Y, 286E, 308P, 428I, 434Y	1.20 × 10^−8^
**RFR 3mut**		
**Variant #**	**Mutations**	**K_D_**
22,717	L309Q, N434D, Y436L	3.14 × 10^−8^
27,638	H310N, H435G, Y436L	3.72 × 10^−8^
21,828	M252R, H310E, H433N	4.01 × 10^−8^
23,131	H310E, N434L, Y436K	4.03 × 10^−8^
26,871	H310R, N434F, Y436K	4.18 × 10^−8^
22,177	K288G, H310G, N434W	5.50 × 10^−8^
27,282	Q311R, M428F, N434F	6.17 × 10^−8^
21,781	K288G, H310G, H433T	6.34 × 10^−8^
23,175	M252W, I253D, N286R	7.10 × 10^−8^
25,334	M252R, K288S, H433S	7.47 × 10^−8^
20,956	M252R, I253G, H433G	8.59 × 10^−8^
26,312	I253D, H433A, Y436K	8.70 × 10^−8^
25,958	K288A, H310T, N434H	8.87 × 10^−8^
23,441	Q311R, M428N, N434F	9.07 × 10^−8^
28,117	K288R, H310D, H433Y	9.16 × 10^−8^
27,621	T256W, H435G, Y436N	9.43 × 10^−8^
20,844	P257V, N286D, T307Y	9.79 × 10^−8^
27,672	M252Q, T256N, H433Y	9.80 × 10^−8^
20,219	M252K, T256Y, H433N	1.01 × 10^−7^
22,805	N286R, T307R, Y436I	1.02 × 10^−7^
**MLP 3mut**		
**Variant #**	**Mutations**	**K_D_**
25,115	L309Q, N434D, Y436L	9.91 × 10^−9^
23,790	H310N, H435G, Y436L	1.18 × 10^−8^
27,256	M252R, H310E, H433N	1.30 × 10^−8^
27,568	H310E, N434L, Y436K	1.36 × 10^−8^
27,086	H310R, N434F, Y436K	1.40 × 10^−8^
22,280	K288G, H310G, N434W	1.42 × 10^−8^
21,044	Q311R, M428F, N434F	1.49 × 10^−8^
20,905	K288G, H310G, H433T	1.61 × 10^−8^
21,807	M252W, I253D, N286R	1.64 × 10^−8^
22,937	M252R, K288S, H433S	1.67 × 10^−8^
22,638	M252R, I253G, H433G	1.71 × 10^−8^
20,841	I253D, H433A, Y436K	1.74 × 10^−8^
25,914	K288A, H310T, N434H	1.76 × 10^−8^
21,608	Q311R, M428N, N434F	1.77 × 10^−8^
27,445	K288R, H310D, H433Y	1.79 × 10^−8^
26,619	T256W, H435G, Y436N	1.79 × 10^−8^
21,178	P257V, N286D, T307Y	1.83 × 10^−8^
24,744	M252Q, T256N, H433Y	1.84 × 10^−8^
21,756	M252K, T256Y, H433N	1.85 × 10^−8^
21,891	N286R, T307R, Y436I	1.85 × 10^−8^
**MLR 3mut**		
**Variant #**	**Mutations**	**K_D_**
23,046	L309Q, N434D, Y436L	8.56 × 10^−10^
23,821	H310N, H435G, Y436L	1.90 × 10^−9^
20,005	M252R, H310E, H433N	1.90 × 10^−9^
21,325	H310E, N434L, Y436K	2.65 × 10^−9^
20,606	H310R, N434F, Y436K	2.72 × 10^−9^
25,146	K288G, H310G, N434W	3.56 × 10^−9^
23,660	Q311R, M428F, N434F	3.96 × 10^−9^
23,971	K288G, H310G, H433T	4.53 × 10^−9^
26,091	M252W, I253D, N286R	4.68 × 10^−9^
27,856	M252R, K288S, H433S	4.85 × 10^−9^
25,298	M252R, I253G, H433G	5.72 × 10^−9^
28,072	I253D, H433A, Y436K	5.80 × 10^−9^
28,288	K288A, H310T, N434H	5.88 × 10^−9^
27,058	Q311R, M428N, N434F	6.28 × 10^−9^
21,705	K288R, H310D, H433Y	6.42 × 10^−9^
25,221	T256W, H435G, Y436N	6.85 × 10^−9^
22,944	P257V, N286D, T307Y	6.95 × 10^−9^
22,506	M252Q, T256N, H433Y	7.37 × 10^−9^
25,804	M252K, T256Y, H433N	7.59 × 10^−9^
27,795	N286R, T307R, Y436I	7.70 × 10^−9^
**SVR 3mut**		
**Variant #**	**Mutations**	**K_D_**
22,166	L309Q, N434D, Y436L	1.09 × 10^−7^
25,716	H310N, H435G, Y436L	1.16 × 10^−7^
26,932	M252R, H310E, H433N	1.26 × 10^−7^
20,339	H310E, N434L, Y436K	1.42 × 10^−7^
26,518	H310R, N434F, Y436K	1.44 × 10^−7^
27,880	K288G, H310G, N434W	1.46 × 10^−7^
21,576	Q311R, M428F, N434F	1.51 × 10^−7^
27,672	K288G, H310G, H433T	1.51 × 10^−7^
22,597	M252W, I253D, N286R	1.58 × 10^−7^
20,333	M252R, K288S, H433S	1.64 × 10^−7^
22,168	M252R, I253G, H433G	1.64 × 10^−7^
23,757	I253D, H433A, Y436K	1.69 × 10^−7^
21,145	K288A, H310T, N434H	1.70 × 10^−7^
20,273	Q311R, M428N, N434F	1.71 × 10^−7^
27,350	K288R, H310D, H433Y	1.72 × 10^−7^
22,409	T256W, H435G, Y436N	1.73 × 10^−7^
24,464	P257V, N286D, T307Y	1.73 × 10^−7^
26,610	M252Q, T256N, H433Y	1.74 × 10^−7^
27,113	M252K, T256Y, H433N	1.76 × 10^−7^
23,602	N286R, T307R, Y436I	1.77 × 10^−7^
**RFR 5mut**		
**Variant #**	**Mutations**	**K_D_**
31,995	M252K, H285Q, T307D, L309K, H433Y	1.24 × 10^−8^
32,898	M252R, N286Q, H310R, M428I, H435N	1.49 × 10^−8^
31,966	M252W, I253H, N286D, Q311K, H433P	1.67 × 10^−8^
37,526	L251I, L309K, H433N, N434Q, Y436L	1.72 × 10^−8^
36,771	I253F, N286R, T307Q, M428F, H435E	1.85 × 10^−8^
33,965	L251A, M252K, L309K, L314S, H433Y	1.94 × 10^−8^
37,863	L309K, Q311E, H433G, N434H, Y436R	2.09 × 10^−8^
32,948	S254N, T307A, L309K, H433T, Y436R	2.45 × 10^−8^
30,050	T256E, N286Q, Q311N, N434H, Y436N	2.53 × 10^−8^
35,099	I253D, P257S, Q311E, M428Y, H433D	2.64 × 10^−8^
38,056	N286E, L309D, L314H, H435N, Y436N	2.90 × 10^−8^
34,584	L251D, M252Q, N286D, V308A, H433A	2.97 × 10^−8^
32,714	M252W, S254T, V308F, H310R, H435S	2.98 × 10^−8^
35,697	M252K, N286R, L309Q, H435N, Y436F	3.14 × 10^−8^
31,821	L251H, M252Q, N286H, Q311N, H433A	3.34 × 10^−8^
37,707	T256S, Q311K, L314R, M428W, N434W	3.44 × 10^−8^
38,325	K288T, V308A, L309K, H433G, Y436K	3.68 × 10^−8^
30,121	I253W, N286Y, L309V, M428F, H433D	3.72 × 10^−8^
32,551	M252F, P257W, H285E, Q311E, N434H	3.84 × 10^−8^
34,867	H285E, N286Y, L309Y, M428H, H433Y	3.88 × 10^−8^
**MLP 5mut**		
**Variant #**	**Mutations**	**K_D_**
36,622	M252K, H285Q, T307D, L309K, H433Y	5.13 × 10^−9^
38,413	M252R, N286Q, H310R, M428I, H435N	7.18 × 10^−9^
32,294	M252W, I253H, N286D, Q311K, H433P	7.20 × 10^−9^
34,399	L251I, L309K, H433N, N434Q, Y436L	7.91 × 10^−9^
35,394	I253F, N286R, T307Q, M428F, H435E	8.58 × 10^−9^
34,608	L251A, M252K, L309K, L314S, H433Y	8.68 × 10^−9^
31,958	L309K, Q311E, H433G, N434H, Y436R	8.91 × 10^−9^
34,236	S254N, T307A, L309K, H433T, Y436R	9.17 × 10^−9^
36,343	T256E, N286Q, Q311N, N434H, Y436N	9.26 × 10^−9^
35,234	I253D, P257S, Q311E, M428Y, H433D	9.47 × 10^−9^
38,030	N286E, L309D, L314H, H435N, Y436N	9.53 × 10^−9^
30,188	L251D, M252Q, N286D, V308A, H433A	9.83 × 10^−9^
35,109	M252W, S254T, V308F, H310R, H435S	9.98 × 10^−9^
32,632	M252K, N286R, L309Q, H435N, Y436F	1.00 × 10^−8^
30,914	L251H, M252Q, N286H, Q311N, H433A	1.04 × 10^−8^
35,398	T256S, Q311K, L314R, M428W, N434W	1.05 × 10^−8^
32,539	K288T, V308A, L309K, H433G, Y436K	1.05 × 10^−8^
35,860	I253W, N286Y, L309V, M428F, H433D	1.06 × 10^−8^
35,943	M252F, P257W, H285E, Q311E, N434H	1.07 × 10^−8^
37,387	H285E, N286Y, L309Y, M428H, H433Y	1.07 × 10^−8^
**MLR 5mut**		
**Variant #**	**Mutations**	**K_D_**
36,120	M252K, H285Q, T307D, L309K, H433Y	4.79 × 10^−10^
31,116	M252R, N286Q, H310R, M428I, H435N	5.14 × 10^−10^
37,434	M252W, I253H, N286D, Q311K, H433P	1.40 × 10^−9^
33,162	L251I, L309K, H433N, N434Q, Y436L	1.44 × 10^−9^
35,517	I253F, N286R, T307Q, M428F, H435E	1.66 × 10^−9^
37,684	L251A, M252K, L309K, L314S, H433Y	1.88 × 10^−9^
37,301	L309K, Q311E, H433G, N434H, Y436R	1.88 × 10^−9^
30,930	S254N, T307A, L309K, H433T, Y436R	1.94 × 10^−9^
36,097	T256E, N286Q, Q311N, N434H, Y436N	1.95 × 10^−9^
38,430	I253D, P257S, Q311E, M428Y, H433D	1.97 × 10^−9^
38,202	N286E, L309D, L314H, H435N, Y436N	2.09 × 10^−9^
37,863	L251D, M252Q, N286D, V308A, H433A	2.09 × 10^−9^
30,545	M252W, S254T, V308F, H310R, H435S	2.15 × 10^−9^
31,317	M252K, N286R, L309Q, H435N, Y436F	2.25 × 10^−9^
34,813	L251H, M252Q, N286H, Q311N, H433A	2.34 × 10^−9^
36,045	T256S, Q311K, L314R, M428W, N434W	2.40 × 10^−9^
38,596	K288T, V308A, L309K, H433G, Y436K	2.55 × 10^−9^
33,006	I253W, N286Y, L309V, M428F, H433D	2.69 × 10^−9^
33,288	M252F, P257W, H285E, Q311E, N434H	2.72 × 10^−9^
33,871	H285E, N286Y, L309Y, M428H, H433Y	2.75 × 10^−9^
**SVR 5mut**		
**Variant #**	**Mutations**	**K_D_**
31,131	M252K, H285Q, T307D, L309K, H433Y	6.12 × 10^−8^
37,573	M252R, N286Q, H310R, M428I, H435N	8.12 × 10^−8^
34,132	M252W, I253H, N286D, Q311K, H433P	8.81 × 10^−8^
32,677	L251I, L309K, H433N, N434Q, Y436L	9.49 × 10^−8^
38,342	I253F, N286R, T307Q, M428F, H435E	1.10 × 10^−7^
31,134	L251A, M252K, L309K, L314S, H433Y	1.12 × 10^−7^
37,613	L309K, Q311E, H433G, N434H, Y436R	1.23 × 10^−7^
36,014	S254N, T307A, L309K, H433T, Y436R	1.29 × 10^−7^
32,967	T256E, N286Q, Q311N, N434H, Y436N	1.48 × 10^−7^
30,390	I253D, P257S, Q311E, M428Y, H433D	1.52 × 10^−7^
31,621	N286E, L309D, L314H, H435N, Y436N	1.58 × 10^−7^
30,551	L251D, M252Q, N286D, V308A, H433A	1.63 × 10^−7^
32,946	M252W, S254T, V308F, H310R, H435S	1.68 × 10^−7^
32,204	M252K, N286R, L309Q, H435N, Y436F	1.74 × 10^−7^
30,254	L251H, M252Q, N286H, Q311N, H433A	1.75 × 10^−7^
31,168	T256S, Q311K, L314R, M428W, N434W	1.77 × 10^−7^
32,902	K288T, V308A, L309K, H433G, Y436K	1.78 × 10^−7^
30,243	I253W, N286Y, L309V, M428F, H433D	1.79 × 10^−7^
30,417	M252F, P257W, H285E, Q311E, N434H	1.83 × 10^−7^
30,211	H285E, N286Y, L309Y, M428H, H433Y	1.84 × 10^−7^
**RFR 8mut**		
**Variant #**	**Mutations**	**K_D_**
30,849	M252W, T256P, N286K, L309K, Q311A, M428F, Y436G	5.67 × 10^−9^
30,198	L251R, I253T, H285N, N286D, L309K, M428F, N434D	1.21 × 10^−8^
30,864	L251T, M252Y, I253P, N286K, V308F, L309R, H433G	1.52 × 10^−8^
30,501	M252Y, I253E, H285I, N286D, V308A, N434H	1.73 × 10^−8^
30,454	M252Y, N286Q, K288F, L309W, Q311L, N434Y	1.77 × 10^−8^
30,390	R255Y, P257N, H285D, V308A, L309K, M428W, N434H	1.94 × 10^−8^
30,947	M252W, I253Y, R255F, N286E, L309D, Q311K, H433P	2.32 × 10^−8^
30,169	L251T, I253D, R255S, T256S, Q311A, M428F, H433G	2.60 × 10^−8^
30,358	M252E, P257V, L309K, M428L, H433F, H435K	2.85 × 10^−8^
30,582	P257A, H285I, T307W, M428W, N434H	2.85 × 10^−8^
30,338	R255Q, N286K, T307Q, L309P, M428F, H433I, H435R	3.57 × 10^−8^
30,211	H285E, N286K, T307R, L309E, Q311K, M428W, N434F	3.59 × 10^−8^
30,974	I253S, T256S, H285D, N286E, V308A, M428L, H435E	3.94 × 10^−8^
30,696	M252D, N286W, L309R, Q311V, N434H, H435K	4.33 × 10^−8^
30,401	M252W, I253D, P257A, V308F, L309E, N434W	4.42 × 10^−8^
30,416	I253P, T256V, N286R, Q311K, M428W, N434F	4.79 × 10^−8^
30,777	M252W, P257T, N286H, T307F, L309G, H433L	5.22 × 10^−8^
30,280	M252V, R255Q, T256N, N286H, M428F, N434Y, Y436K	5.47 × 10^−8^
30,913	L251P, T256P, N286Q, L309K, Q311I, Y436G	5.66 × 10^−8^
30,948	L251G, T256N, N286E, V308A, L309K, H433L, N434T	5.66 × 10^−8^
**MLP 8mut**		
**Variant #**	**Mutations**	**K_D_**
30,126	M252W, T256P, N286K, L309K, Q311A, M428F, Y436G	5.75 × 10^−9^
30,501	L251R, I253T, H285N, N286D, L309K, M428F, N434D	6.73 × 10^−9^
30,603	L251T, M252Y, I253P, N286K, V308F, L309R, H433G	7.09 × 10^−9^
30,947	M252Y, I253E, H285I, N286D, V308A, N434H	8.43 × 10^−9^
30,070	M252Y, N286Q, K288F, L309W, Q311L, N434Y	9.86 × 10^−9^
30,582	R255Y, P257N, H285D, V308A, L309K, M428W, N434H	1.09 × 10^−8^
30,198	M252W, I253Y, R255F, N286E, L309D, Q311K, H433P	1.14 × 10^−8^
30,822	L251T, I253D, R255S, T256S, Q311A, M428F, H433G	1.18 × 10^−8^
30,554	M252E, P257V, L309K, M428L, H433F, H435K	1.37 × 10^−8^
30,950	P257A, H285I, T307W, M428W, N434H	1.40 × 10^−8^
30,154	R255Q, N286K, T307Q, L309P, M428F, H433I, H435R	1.46 × 10^−8^
30,942	H285E, N286K, T307R, L309E, Q311K, M428W, N434F	1.47 × 10^−8^
30,842	I253S, T256S, H285D, N286E, V308A, M428L, H435E	1.49 × 10^−8^
30,454	M252D, N286W, L309R, Q311V, N434H, H435K	1.52 × 10^−8^
30,259	M252W, I253D, P257A, V308F, L309E, N434W	1.55 × 10^−8^
30,042	I253P, T256V, N286R, Q311K, M428W, N434F	1.59 × 10^−8^
30,782	M252W, P257T, N286H, T307F, L309G, H433L	1.60 × 10^−8^
30,241	M252V, R255Q, T256N, N286H, M428F, N434Y, Y436K	1.62 × 10^−8^
30,171	L251P, T256P, N286Q, L309K, Q311I, Y436G	1.64 × 10^−8^
30,365	L251G, T256N, N286E, V308A, L309K, H433L, N434T	1.68 × 10^−8^
**MLR 8mut**		
**Variant #**	**Mutations**	**K_D_**
30,835	M252W, T256P, N286K, L309K, Q311A, M428F, Y436G	2.34 × 10-11
30,259	L251R, I253T, H285N, N286D, L309K, M428F, N434D	8.60 × 10-11
30,184	L251T, M252Y, I253P, N286K, V308F, L309R, H433G	3.28 × 10^−10^
30,558	M252Y, I253E, H285I, N286D, V308A, N434H	4.17 × 10^−10^
30,317	M252Y, N286Q, K288F, L309W, Q311L, N434Y	5.83 × 10^−10^
30,395	R255Y, P257N, H285D, V308A, L309K, M428W, N434H	6.03 × 10^−10^
30,787	M252W, I253Y, R255F, N286E, L309D, Q311K, H433P	6.18 × 10^−10^
30,968	L251T, I253D, R255S, T256S, Q311A, M428F, H433G	1.34 × 10^−9^
30,762	M252E, P257V, L309K, M428L, H433F, H435K	1.36 × 10^−9^
30,253	P257A, H285I, T307W, M428W, N434H	1.68 × 10^−9^
30,500	R255Q, N286K, T307Q, L309P, M428F, H433I, H435R	2.17 × 10^−9^
30,926	H285E, N286K, T307R, L309E, Q311K, M428W, N434F	2.26 × 10^−9^
30,023	I253S, T256S, H285D, N286E, V308A, M428L, H435E	2.27 × 10^−9^
30,515	M252D, N286W, L309R, Q311V, N434H, H435K	2.49 × 10^−9^
30,087	M252W, I253D, P257A, V308F, L309E, N434W	2.51 × 10^−9^
30,209	I253P, T256V, N286R, Q311K, M428W, N434F	3.25 × 10^−9^
30,832	M252W, P257T, N286H, T307F, L309G, H433L	3.36 × 10^−9^
30,947	M252V, R255Q, T256N, N286H, M428F, N434Y, Y436K	3.49 × 10^−9^
30,179	L251P, T256P, N286Q, L309K, Q311I, Y436G	3.51 × 10^−9^
30,577	L251G, T256N, N286E, V308A, L309K, H433L, N434T	3.74 × 10^−9^
**SVR 8mut**		
**Variant #**	**Mutations**	**K_D_**
30,401	M252W, T256P, N286K, L309K, Q311A, M428F, Y436G	1.53 × 10^−7^
30,245	L251R, I253T, H285N, N286D, L309K, M428F, N434D	1.71 × 10^−7^
30,105	L251T, M252Y, I253P, N286K, V308F, L309R, H433G	1.84 × 10^−7^
30,625	M252Y, I253E, H285I, N286D, V308A, N434H	1.91 × 10^−7^
30,022	M252Y, N286Q, K288F, L309W, Q311L, N434Y	1.92 × 10^−7^
30,142	R255Y, P257N, H285D, V308A, L309K, M428W, N434H	1.96 × 10^−7^
30,501	M252W, I253Y, R255F, N286E, L309D, Q311K, H433P	2.02 × 10^−7^
30,097	L251T, I253D, R255S, T256S, Q311A, M428F, H433G	2.03 × 10^−7^
30,974	M252E, P257V, L309K, M428L, H433F, H435K	2.04 × 10^−7^
30,684	P257A, H285I, T307W, M428W, N434H	2.04 × 10^−7^
30,186	R255Q, N286K, T307Q, L309P, M428F, H433I, H435R	2.04 × 10^−7^
30,955	H285E, N286K, T307R, L309E, Q311K, M428W, N434F	2.04 × 10^−7^
30,582	I253S, T256S, H285D, N286E, V308A, M428L, H435E	2.05 × 10^−7^
30,012	M252D, N286W, L309R, Q311V, N434H, H435K	2.05 × 10^−7^
30,905	M252W, I253D, P257A, V308F, L309E, N434W	2.05 × 10^−7^
30,502	I253P, T256V, N286R, Q311K, M428W, N434F	2.05 × 10^−7^
30,785	M252W, P257T, N286H, T307F, L309G, H433L	2.05 × 10^−7^
30,685	M252V, R255Q, T256N, N286H, M428F, N434Y, Y436K	2.06 × 10^−7^
30,261	L251P, T256P, N286Q, L309K, Q311I, Y436G	2.06 × 10^−7^
30,206	L251G, T256N, N286E, V308A, L309K, H433L, N434T	2.06 × 10^−7^
**Model FLS**		
**RFR Learning_Set**		
**Variant #**	**Mutations**	**K_D_**
833	235R, 239K, 252Y, 286E, 307Q, 308P, 311A, 428I, 434Y, 436V	3.66 × 10^−9^
831	250V, 252Y, 286E, 307Q, 308P, 311A, 428I, 434Y, 436V	3.79 × 10^−9^
832	235R, 239K, 250V, 252Y, 286E, 307Q, 308P, 311A, 428I, 434Y, 436V	3.92 × 10^−9^
802	235R, 239K, 252Y, 286E, 307Q, 308P, 311A, 434Y, 436V	4.09 × 10^−9^
829	235K, 239K, 252Y, 286E, 307Q, 308P, 311A, 434Y, 436V	4.09 × 10^−9^
800	250V, 252Y, 286E, 307Q, 308P, 311A, 434Y, 436V	4.24 × 10^−9^
801	235R, 239K, 250V, 252Y, 286E, 307Q, 308P, 311A, 434Y, 436V	4.39 × 10^−9^
828	235K, 239K, 250V, 252Y, 286E, 307Q, 308P, 311A, 434Y, 436V	4.39 × 10^−9^
568	252Y, 286E, 307Q, 308P, 311A, 428I, 434Y	4.71 × 10^−9^
1	239K, 252Y, 270F, 286E, 307Q, 308P, 311A, 428I, 434Y	5.40 × 10^−9^
567	252Y, 286E, 307Q, 308P, 311A, 434Y	7.07 × 10^−9^
5	239K, 252Y, 270F, 286E, 307Q, 308P, 428I, 434Y	7.27 × 10^−9^
2	239K, 252W, 286E, 308P, 428Y, 434Y	7.34 × 10^−9^
7	239K, 252Y, 270F, 286E, 308P, 387E, 428I, 434Y	7.60 × 10^−9^
527	239K, 252Y, 286E, 307Q, 308P, 311A, 434Y	7.90 × 10^−9^
6	239K, 252Y, 286E, 308P, 428I, 434Y	7.95 × 10^−9^
8	239K, 252Y, 270F, 286E, 308P, 428I, 434Y	8.12 × 10^−9^
4	239K, 252Y, 270F, 286E, 308P, 311A, 428I, 434Y	8.41 × 10^−9^
3	239K, 252W, 256E, 286E, 308P, 428Y, 434Y	8.59 × 10^−9^
565	252Y, 286E, 308P, 428I, 434Y	1.03 × 10^−8^
**MLP learning_set**		
**Variant #**	**Mutations**	**K_D_**
20	235R, 239K, 252Y, 286E, 307Q, 308P, 311A, 428I, 434Y, 436V	2.96 × 10^−8^
90	250V, 252Y, 286E, 307Q, 308P, 311A, 428I, 434Y, 436V	3.42 × 10^−8^
40	235R, 239K, 250V, 252Y, 286E, 307Q, 308P, 311A, 428I, 434Y, 436V	3.42 × 10^−8^
566	235R, 239K, 252Y, 286E, 307Q, 308P, 311A, 434Y, 436V	3.51 × 10^−8^
570	235K, 239K, 252Y, 286E, 307Q, 308P, 311A, 434Y, 436V	3.51 × 10^−8^
569	250V, 252Y, 286E, 307Q, 308P, 311A, 434Y, 436V	3.51 × 10^−8^
204	235R, 239K, 250V, 252Y, 286E, 307Q, 308P, 311A, 434Y, 436V	3.51 × 10^−8^
119	235K, 239K, 250V, 252Y, 286E, 307Q, 308P, 311A, 434Y, 436V	3.51 × 10^−8^
110	252Y, 286E, 307Q, 308P, 311A, 428I, 434Y	3.51 × 10^−8^
44	239K, 252Y, 270F, 286E, 307Q, 308P, 311A, 428I, 434Y	3.51 × 10^−8^
131	252Y, 286E, 307Q, 308P, 311A, 434Y	3.51 × 10^−8^
581	239K, 252Y, 270F, 286E, 307Q, 308P, 428I, 434Y	3.54 × 10^−8^
23	239K, 252W, 286E, 308P, 428Y, 434Y	3.54 × 10^−8^
19	239K, 252Y, 270F, 286E, 308P, 387E, 428I, 434Y	3.55 × 10^−8^
568	239K, 252Y, 286E, 307Q, 308P, 311A, 434Y	3.57 × 10^−8^
1	239K, 252Y, 286E, 308P, 428I, 434Y	3.57 × 10^−8^
5	239K, 252Y, 270F, 286E, 308P, 428I, 434Y	3.59 × 10^−8^
53	239K, 252Y, 270F, 286E, 308P, 311A, 428I, 434Y	3.80 × 10^−8^
98	239K, 252W, 256E, 286E, 308P, 428Y, 434Y	3.80 × 10^−8^
59	252Y, 286E, 308P, 428I, 434Y	3.84 × 10^−8^
**MLR learning_set**		
**Variant #**	**Mutations**	**K_D_**
684	235R, 239K, 252Y, 286E, 307Q, 308P, 311A, 428I, 434Y, 436V	9.96 × 10^−9^
163	250V, 252Y, 286E, 307Q, 308P, 311A, 428I, 434Y, 436V	1.79 × 10^−8^
167	235R, 239K, 250V, 252Y, 286E, 307Q, 308P, 311A, 428I, 434Y, 436V	2.03 × 10^−8^
216	235R, 239K, 252Y, 286E, 307Q, 308P, 311A, 434Y, 436V	2.03 × 10^−8^
182	235K, 239K, 252Y, 286E, 307Q, 308P, 311A, 434Y, 436V	2.55 × 10^−8^
128	250V, 252Y, 286E, 307Q, 308P, 311A, 434Y, 436V	2.60 × 10^−8^
94	235R, 239K, 250V, 252Y, 286E, 307Q, 308P, 311A, 434Y, 436V	2.64 × 10^−8^
231	235K, 239K, 250V, 252Y, 286E, 307Q, 308P, 311A, 434Y, 436V	2.64 × 10^−8^
120	252Y, 286E, 307Q, 308P, 311A, 428I, 434Y	2.68 × 10^−8^
495	239K, 252Y, 270F, 286E, 307Q, 308P, 311A, 428I, 434Y	2.69 × 10^−8^
24	252Y, 286E, 307Q, 308P, 311A, 434Y	2.69 × 10^−8^
192	239K, 252Y, 270F, 286E, 307Q, 308P, 428I, 434Y	2.84 × 10^−8^
127	239K, 252W, 286E, 308P, 428Y, 434Y	2.84 × 10^−8^
145	239K, 252Y, 270F, 286E, 308P, 387E, 428I, 434Y	2.85 × 10^−8^
496	239K, 252Y, 286E, 307Q, 308P, 311A, 434Y	2.89 × 10^−8^
235	239K, 252Y, 286E, 308P, 428I, 434Y	2.89 × 10^−8^
130	239K, 252Y, 270F, 286E, 308P, 428I, 434Y	2.89 × 10^−8^
77	239K, 252Y, 270F, 286E, 308P, 311A, 428I, 434Y	2.89 × 10^−8^
82	239K, 252W, 256E, 286E, 308P, 428Y, 434Y	2.89 × 10^−8^
107	252Y, 286E, 308P, 428I, 434Y	2.89 × 10^−8^
**SVR learning_set**		
**Variant #**	**Mutations**	**K_D_**
243	235R, 239K, 252Y, 286E, 307Q, 308P, 311A, 428I, 434Y, 436V	7.25 × 10^−9^
276	250V, 252Y, 286E, 307Q, 308P, 311A, 428I, 434Y, 436V	7.26 × 10^−9^
208	235R, 239K, 250V, 252Y, 286E, 307Q, 308P, 311A, 428I, 434Y, 436V	1.29 × 10^−8^
8	235R, 239K, 252Y, 286E, 307Q, 308P, 311A, 434Y, 436V	1.31 × 10^−8^
7	235K, 239K, 252Y, 286E, 307Q, 308P, 311A, 434Y, 436V	1.31 × 10^−8^
6	250V, 252Y, 286E, 307Q, 308P, 311A, 434Y, 436V	1.31 × 10^−8^
565	235R, 239K, 250V, 252Y, 286E, 307Q, 308P, 311A, 434Y, 436V	1.31 × 10^−8^
4	235K, 239K, 250V, 252Y, 286E, 307Q, 308P, 311A, 434Y, 436V	1.31 × 10^−8^
800	252Y, 286E, 307Q, 308P, 311A, 428I, 434Y	1.34 × 10^−8^
828	239K, 252Y, 270F, 286E, 307Q, 308P, 311A, 428I, 434Y	1.34 × 10^−8^
801	252Y, 286E, 307Q, 308P, 311A, 434Y	1.34 × 10^−8^
802	239K, 252Y, 270F, 286E, 307Q, 308P, 428I, 434Y	1.34 × 10^−8^
829	239K, 252W, 286E, 308P, 428Y, 434Y	1.34 × 10^−8^
633	239K, 252Y, 270F, 286E, 308P, 387E, 428I, 434Y	1.36 × 10^−8^
59	239K, 252Y, 286E, 307Q, 308P, 311A, 434Y	1.42 × 10^−8^
43	239K, 252Y, 286E, 308P, 428I, 434Y	1.67 × 10^−8^
38	239K, 252Y, 270F, 286E, 308P, 428I, 434Y	1.67 × 10^−8^
37	239K, 252Y, 270F, 286E, 308P, 311A, 428I, 434Y	1.67 × 10^−8^
42	239K, 252W, 256E, 286E, 308P, 428Y, 434Y	1.67 × 10^−8^
41	252Y, 286E, 308P, 428I, 434Y	1.67 × 10^−8^
**RFR 3mut**		
**Variant #**	**Mutations**	**K_D_**
24,936	H310L, N434H, Y436Q	3.32 × 10^−8^
23,681	T307K, H310D, N434F	3.80 × 10^−8^
22,900	P257H, H310G, N434F	4.03 × 10^−8^
22,177	K288G, H310G, N434W	4.59 × 10^−8^
24,569	H310V, Q311Y, N434F	4.84 × 10^−8^
23,303	H310D, H433S, N434F	4.85 × 10^−8^
24,652	H310E, Q311G, N434F	4.88 × 10^−8^
22,597	H285W, H310E, N434W	4.98 × 10^−8^
20,285	P257I, T307G, N434F	5.05 × 10^−8^
23,152	I253E, H310A, N434W	5.38 × 10^−8^
27,018	H310A, M428K, N434F	6.27 × 10^−8^
25,958	K288A, H310T, N434H	6.53 × 10^−8^
26,256	R255K, T307K, N434F	8.46 × 10^−8^
23,826	S254A, T307D, N434F	9.36 × 10^−8^
26,389	R255K, T307F, N434W	9.95 × 10^−8^
21,973	H285T, L309N, N434F	1.04 × 10^−7^
27,024	M252F, L309D, N434H	1.17 × 10^−7^
26,949	T256L, V308P, N434W	1.26 × 10^−7^
20,052	L309G, M428L, N434F	1.27 × 10^−7^
23,029	R255Y, T307S, N434H	1.28 × 10^−7^
**MLP 3mut**		
**Variant #**	**Mutations**	**K_D_**
20,285	H310L, N434H, Y436Q	2.53 × 10^−8^
23,242	T307K, H310D, N434F	2.71 × 10^−8^
26,256	P257H, H310G, N434F	3.19 × 10^−8^
22,900	K288G, H310G, N434W	3.77 × 10^−8^
20,052	H310V, Q311Y, N434F	4.56 × 10^−8^
26,389	H310D, H433S, N434F	5.99 × 10^−8^
23,681	H310E, Q311G, N434F	6.05 × 10^−8^
22,715	H285W, H310E, N434W	6.82 × 10^−8^
23,826	P257I, T307G, N434F	6.94 × 10^−8^
21,576	I253E, H310A, N434W	7.11 × 10^−8^
20,293	H310A, M428K, N434F	7.80 × 10^−8^
21,093	K288A, H310T, N434H	8.15 × 10^−8^
22,166	R255K, T307K, N434F	8.20 × 10^−8^
21,460	S254A, T307D, N434F	9.15 × 10^−8^
27,689	R255K, T307F, N434W	1.10 × 10^−7^
20,625	H285T, L309N, N434F	1.12 × 10^−7^
26,125	M252F, L309D, N434H	1.15 × 10^−7^
22,533	T256L, V308P, N434W	1.15 × 10^−7^
26,616	L309G, M428L, N434F	1.16 × 10^−7^
20,670	R255Y, T307S, N434H	1.21 × 10^−7^
**MLR 3mut**		
**Variant #**	**Mutations**	**K_D_**
20,285	H310L, N434H, Y436Q	2.00 × 10^−8^
23,242	T307K, H310D, N434F	2.28 × 10^−8^
26,256	P257H, H310G, N434F	3.15 × 10^−8^
22,900	K288G, H310G, N434W	3.73 × 10^−8^
21,460	H310V, Q311Y, N434F	5.02 × 10^−8^
20,052	H310D, H433S, N434F	5.07 × 10^−8^
23,681	H310E, Q311G, N434F	5.34 × 10^−8^
23,826	H285W, H310E, N434W	6.64 × 10^−8^
20,625	P257I, T307G, N434F	6.95 × 10^−8^
26,616	I253E, H310A, N434W	7.54 × 10^−8^
20,293	H310A, M428K, N434F	7.62 × 10^−8^
21,093	K288A, H310T, N434H	7.68 × 10^−8^
23,441	R255K, T307K, N434F	7.70 × 10^−8^
27,689	S254A, T307D, N434F	8.09 × 10^−8^
25,977	R255K, T307F, N434W	8.61 × 10^−8^
21,576	H285T, L309N, N434F	8.84 × 10^−8^
26,389	M252F, L309D, N434H	8.84 × 10^−8^
22,715	T256L, V308P, N434W	9.07 × 10^−8^
22,166	L309G, M428L, N434F	9.34 × 10^−8^
27,485	R255Y, T307S, N434H	1.02 × 10^−7^
**SVR 3mut**		
**Variant #**	**Mutations**	**K_D_**
22,166	H310L, N434H, Y436Q	3.15 × 10^−8^
22,050	T307K, H310D, N434F	3.63 × 10^−8^
26,109	P257H, H310G, N434F	5.30 × 10^−8^
20,052	K288G, H310G, N434W	1.15 × 10^−7^
23,242	H310V, Q311Y, N434F	1.21 × 10^−7^
23,889	H310D, H433S, N434F	1.28 × 10^−7^
25,411	H310E, Q311G, N434F	1.40 × 10^−7^
22,409	H285W, H310E, N434W	1.66 × 10^−7^
25,263	P257I, T307G, N434F	1.69 × 10^−7^
21,432	I253E, H310A, N434W	1.70 × 10^−7^
22,743	H310A, M428K, N434F	1.78 × 10^−7^
22,378	K288A, H310T, N434H	1.82 × 10^−7^
20,285	R255K, T307K, N434F	1.88 × 10^−7^
23,826	S254A, T307D, N434F	1.93 × 10^−7^
24,481	R255K, T307F, N434W	2.00 × 10^−7^
21,447	H285T, L309N, N434F	2.01 × 10^−7^
23,303	M252F, L309D, N434H	2.05 × 10^−7^
28,002	T256L, V308P, N434W	2.15 × 10^−7^
26,447	L309G, M428L, N434F	2.32 × 10^−7^
25,009	R255Y, T307S, N434H	2.35 × 10^−7^
**RFR 5mut**		
**Variant #**	**Mutations**	**K_D_**
37,435	S254G, T256Q, H310I, Q311W, N434H	1.07 × 10^−8^
35,309	M252Q, R255K, L309N, N434H, Y436R	1.16 × 10^−8^
35,463	H285Y, N286R, H310N, H433Y, N434H	1.36 × 10^−8^
37,379	S254T, K288G, H310S, M428V, N434F	2.91 × 10^−8^
38,616	M252R, P257E, T307Q, V308L, N434H	4.50 × 10^−8^
31,621	I253E, V308Y, H310D, L314I, N434Y	5.33 × 10^−8^
30,182	I253T, K288N, V308T, H310D, N434W	5.58 × 10^−8^
37,088	R255K, T256Q, V308H, L314S, N434Y	5.96 × 10^−8^
31,104	S254A, V308D, H310I, M428Q, N434H	6.29 × 10^−8^
37,174	M252P, T307Y, V308R, Q311E, N434F	6.62 × 10^−8^
33,328	S254G, H285S, N286F, L309A, N434Y	6.77 × 10^−8^
33,978	M252F, R255A, N286R, H310N, N434H	7.29 × 10^−8^
31,320	V308I, L309N, M428S, N434W, H435D	8.29 × 10^−8^
33,091	R255K, T307W, Q311D, H433Q, N434F	8.31 × 10^−8^
31,232	I253P, P257G, T307E, L309Y, N434H	8.36 × 10^−8^
33,342	I253D, S254D, T256V, T307G, N434F	8.43 × 10^−8^
33,591	M252T, I253V, N286G, H310L, N434H	8.67 × 10^−8^
30,984	P257K, T307A, V308F, M428H, N434Y	8.69 × 10^−8^
30,585	M252T, P257H, V308Y, L309F, N434F	8.74 × 10^−8^
38,371	M252E, S254F, P257Y, T307A, N434H	8.88 × 10^−8^
**MLP 5mut**		
**Variant #**	**Mutations**	**K_D_**
33,091	S254G, T256Q, H310I, Q311W, N434H	3.22 × 10^−8^
37,254	M252Q, R255K, L309N, N434H, Y436R	3.28 × 10^−8^
33,646	H285Y, N286R, H310N, H433Y, N434H	3.42 × 10^−8^
34,469	S254T, K288G, H310S, M428V, N434F	4.33 × 10^−8^
34,320	M252R, P257E, T307Q, V308L, N434H	4.47 × 10^−8^
32,501	I253E, V308Y, H310D, L314I, N434Y	4.61 × 10^−8^
34,132	I253T, K288N, V308T, H310D, N434W	5.00 × 10^−8^
30,984	R255K, T256Q, V308H, L314S, N434Y	5.02 × 10^−8^
32,098	S254A, V308D, H310I, M428Q, N434H	5.06 × 10^−8^
34,494	M252P, T307Y, V308R, Q311E, N434F	5.83 × 10^−8^
34,889	S254G, H285S, N286F, L309A, N434Y	5.97 × 10^−8^
33,342	M252F, R255A, N286R, H310N, N434H	6.06 × 10^−8^
31,505	V308I, L309N, M428S, N434W, H435D	6.15 × 10^−8^
35,586	R255K, T307W, Q311D, H433Q, N434F	6.60 × 10^−8^
37,174	I253P, P257G, T307E, L309Y, N434H	6.85 × 10^−8^
31,465	I253D, S254D, T256V, T307G, N434F	7.08 × 10^−8^
36,149	M252T, I253V, N286G, H310L, N434H	7.21 × 10^−8^
33,080	P257K, T307A, V308F, M428H, N434Y	7.22 × 10^−8^
37,661	M252T, P257H, V308Y, L309F, N434F	7.23 × 10^−8^
30,906	M252E, S254F, P257Y, T307A, N434H	7.32 × 10^−8^
**MLR 5mut**		
**Variant #**	**Mutations**	**K_D_**
30,906	S254G, T256Q, H310I, Q311W, N434H	1.22 × 10^−8^
37,580	M252Q, R255K, L309N, N434H, Y436R	1.33 × 10^−8^
33,885	H285Y, N286R, H310N, H433Y, N434H	1.39 × 10^−8^
32,098	S254T, K288G, H310S, M428V, N434F	1.75 × 10^−8^
33,646	M252R, P257E, T307Q, V308L, N434H	3.23 × 10^−8^
34,320	I253E, V308Y, H310D, L314I, N434Y	3.36 × 10^−8^
34,469	I253T, K288N, V308T, H310D, N434W	3.62 × 10^−8^
33,091	R255K, T256Q, V308H, L314S, N434Y	3.74 × 10^−8^
37,954	S254A, V308D, H310I, M428Q, N434H	4.06 × 10^−8^
30,984	M252P, T307Y, V308R, Q311E, N434F	4.59 × 10^−8^
37,254	S254G, H285S, N286F, L309A, N434Y	4.88 × 10^−8^
34,889	M252F, R255A, N286R, H310N, N434H	5.60 × 10^−8^
33,342	V308I, L309N, M428S, N434W, H435D	5.62 × 10^−8^
31,505	R255K, T307W, Q311D, H433Q, N434F	6.39 × 10^−8^
33,080	I253P, P257G, T307E, L309Y, N434H	6.61 × 10^−8^
34,248	I253D, S254D, T256V, T307G, N434F	6.68 × 10^−8^
35,586	M252T, I253V, N286G, H310L, N434H	6.86 × 10^−8^
33,509	P257K, T307A, V308F, M428H, N434Y	6.93 × 10^−8^
37,174	M252T, P257H, V308Y, L309F, N434F	7.15 × 10^−8^
34,132	M252E, S254F, P257Y, T307A, N434H	7.67 × 10^−8^
**SVR 5mut**		
**Variant #**	**Mutations**	**K_D_**
31,465	S254G, T256Q, H310I, Q311W, N434H	9.50 × 10^−9^
33,646	M252Q, R255K, L309N, N434H, Y436R	2.70 × 10^−8^
30,585	H285Y, N286R, H310N, H433Y, N434H	2.83 × 10^−8^
31,612	S254T, K288G, H310S, M428V, N434F	3.98 × 10^−8^
30,423	M252R, P257E, T307Q, V308L, N434H	4.17 × 10^−8^
31,505	I253E, V308Y, H310D, L314I, N434Y	4.66 × 10^−8^
38,216	I253T, K288N, V308T, H310D, N434W	6.22 × 10^−8^
34,132	R255K, T256Q, V308H, L314S, N434Y	7.10 × 10^−8^
32,098	S254A, V308D, H310I, M428Q, N434H	7.50 × 10^−8^
37,379	M252P, T307Y, V308R, Q311E, N434F	9.68 × 10^−8^
33,080	S254G, H285S, N286F, L309A, N434Y	1.03 × 10^−7^
36,338	M252F, R255A, N286R, H310N, N434H	1.03 × 10^−7^
31,469	V308I, L309N, M428S, N434W, H435D	1.14 × 10^−7^
37,661	R255K, T307W, Q311D, H433Q, N434F	1.23 × 10^−7^
36,149	I253P, P257G, T307E, L309Y, N434H	1.30 × 10^−7^
37,777	I253D, S254D, T256V, T307G, N434F	1.37 × 10^−7^
34,998	M252T, I253V, N286G, H310L, N434H	1.37 × 10^−7^
38,029	P257K, T307A, V308F, M428H, N434Y	1.44 × 10^−7^
34,712	M252T, P257H, V308Y, L309F, N434F	1.49 × 10^−7^
32,754	M252E, S254F, P257Y, T307A, N434H	1.51 × 10^−7^
**RFR 8mut**		
**Variant #**	**Mutations**	**K_D_**
30,401	M252W, I253D, P257A, V308F, L309E, N434W	3.06 × 10^−8^
30,320	L251Q, P257S, N286P, V308W, L309E, Q311A, N434H	5.89 × 10^−8^
30,747	M252G, T256A, L309D, N434W, H435E	6.07 × 10^−8^
30,663	I253S, P257V, K288G, T307G, N434H, Y436S	7.74 × 10^−8^
30,083	L251P, P257T, K288N, T307R, V308P, L309K, N434H	8.02 × 10^−8^
30,582	P257A, H285I, T307W, M428W, N434H	9.00 × 10^−8^
30,549	T256G, H285D, T307Y, L309T, N434F, H435T	9.50 × 10^−8^
30,647	M252W, T256P, P257A, K288L, T307S, M428I, N434H	9.59 × 10^−8^
30,596	L251Q, K288F, T307I, L309K, Q311T, M428I, N434W	1.01 × 10^−7^
30,548	L251R, M252H, V308R, L309D, N434H, Y436G	1.06 × 10^−7^
30,915	K288E, T307E, V308N, L309W, M428W, N434Y	1.07 × 10^−7^
30,501	M252Y, I253E, H285I, N286D, V308A, N434H	1.10 × 10^−7^
30,848	M252V, T256A, L309G, H433S, N434W, H435K	1.11 × 10^−7^
30,912	R255S, P257N, H285R, L309D, M428I, N434Y	1.14 × 10^−7^
30,116	M252I, I253S, N434W, H435P, Y436K	1.17 × 10^−7^
30,780	I253T, N286Q, V308P, Q311A, N434Y, Y436S	1.21 × 10^−7^
30,625	T256N, N286L, K288P, T307P, Q311A, M428L, N434Y	1.23 × 10^−7^
30,245	M252E, P257T, H285N, V308P, Q311L, N434Y	1.24 × 10^−7^
30,045	P257Y, Q311T, M428L, N434Y, H435P	1.36 × 10^−7^
30,560	P257A, K288T, T307F, Q311V, N434H, H435K	1.38 × 10^−7^
**MLP 8mut**		
**Variant #**	**Mutations**	**K_D_**
30,829	M252W, I253D, P257A, V308F, L309E, N434W	4.39 × 10^−8^
30,549	L251Q, P257S, N286P, V308W, L309E, Q311A, N434H	4.75 × 10^−8^
30,625	M252G, T256A, L309D, N434W, H435E	6.72 × 10^−8^
30,061	I253S, P257V, K288G, T307G, N434H, Y436S	7.36 × 10^−8^
30,860	L251P, P257T, K288N, T307R, V308P, L309K, N434H	8.15 × 10^−8^
30,721	P257A, H285I, T307W, M428W, N434H	8.68 × 10^−8^
30,045	T256G, H285D, T307Y, L309T, N434F, H435T	9.13 × 10^−8^
30,234	M252W, T256P, P257A, K288L, T307S, M428I, N434H	9.13 × 10^−8^
30,852	L251Q, K288F, T307I, L309K, Q311T, M428I, N434W	9.38 × 10^−8^
30,063	L251R, M252H, V308R, L309D, N434H, Y436G	9.75 × 10^−8^
30,022	K288E, T307E, V308N, L309W, M428W, N434Y	1.09 × 10^−7^
30,565	M252Y, I253E, H285I, N286D, V308A, N434H	1.09 × 10^−7^
30,490	M252V, T256A, L309G, H433S, N434W, H435K	1.10 × 10^−7^
30,669	R255S, P257N, H285R, L309D, M428I, N434Y	1.13 × 10^−7^
30,401	M252I, I253S, N434W, H435P, Y436K	1.13 × 10^−7^
30,583	I253T, N286Q, V308P, Q311A, N434Y, Y436S	1.17 × 10^−7^
30,245	T256N, N286L, K288P, T307P, Q311A, M428L, N434Y	1.21 × 10^−7^
30,211	M252E, P257T, H285N, V308P, Q311L, N434Y	1.26 × 10^−7^
30,596	P257Y, Q311T, M428L, N434Y, H435P	1.42 × 10^−7^
30,683	P257A, K288T, T307F, Q311V, N434H, H435K	1.43 × 10^−7^
**MLR 8mut**		
**Variant #**	**Mutations**	**K_D_**
30,829	M252W, I253D, P257A, V308F, L309E, N434W	2.17 × 10^−8^
30,669	L251Q, P257S, N286P, V308W, L309E, Q311A, N434H	2.84 × 10^−8^
30,549	M252G, T256A, L309D, N434W, H435E	4.85 × 10^−8^
30,583	I253S, P257V, K288G, T307G, N434H, Y436S	7.34 × 10^−8^
30,022	L251P, P257T, K288N, T307R, V308P, L309K, N434H	7.90 × 10^−8^
30,721	P257A, H285I, T307W, M428W, N434H	8.09 × 10^−8^
30,063	T256G, H285D, T307Y, L309T, N434F, H435T	8.84 × 10^−8^
30,100	M252W, T256P, P257A, K288L, T307S, M428I, N434H	1.05 × 10^−7^
30,625	L251Q, K288F, T307I, L309K, Q311T, M428I, N434W	1.05 × 10^−7^
30,401	L251R, M252H, V308R, L309D, N434H, Y436G	1.11 × 10^−7^
30,683	K288E, T307E, V308N, L309W, M428W, N434Y	1.11 × 10^−7^
30,565	M252Y, I253E, H285I, N286D, V308A, N434H	1.12 × 10^−7^
30,225	M252V, T256A, L309G, H433S, N434W, H435K	1.13 × 10^−7^
30,045	R255S, P257N, H285R, L309D, M428I, N434Y	1.24 × 10^−7^
30,605	M252I, I253S, N434W, H435P, Y436K	1.30 × 10^−7^
30,860	I253T, N286Q, V308P, Q311A, N434Y, Y436S	1.36 × 10^−7^
30,061	T256N, N286L, K288P, T307P, Q311A, M428L, N434Y	1.40 × 10^−7^
30,245	M252E, P257T, H285N, V308P, Q311L, N434Y	1.52 × 10^−7^
30,211	P257Y, Q311T, M428L, N434Y, H435P	1.66 × 10^−7^
30,085	P257A, K288T, T307F, Q311V, N434H, H435K	1.67 × 10^−7^
**SVR 8mut**		
**Variant #**	**Mutations**	**K_D_**
30,501	M252W, I253D, P257A, V308F, L309E, N434W	3.20 × 10^−8^
30,401	L251Q, P257S, N286P, V308W, L309E, Q311A, N434H	4.26 × 10^−8^
30,848	M252G, T256A, L309D, N434W, H435E	8.11 × 10^−8^
30,479	I253S, P257V, K288G, T307G, N434H, Y436S	1.19 × 10^−7^
30,829	L251P, P257T, K288N, T307R, V308P, L309K, N434H	1.26 × 10^−7^
30,045	P257A, H285I, T307W, M428W, N434H	1.27 × 10^−7^
30,397	T256G, H285D, T307Y, L309T, N434F, H435T	1.69 × 10^−7^
30,116	M252W, T256P, P257A, K288L, T307S, M428I, N434H	1.70 × 10^−7^
30,157	L251Q, K288F, T307I, L309K, Q311T, M428I, N434W	1.74 × 10^−7^
30,336	L251R, M252H, V308R, L309D, N434H, Y436G	1.86 × 10^−7^
30,061	K288E, T307E, V308N, L309W, M428W, N434Y	1.88 × 10^−7^
30,560	M252Y, I253E, H285I, N286D, V308A, N434H	1.89 × 10^−7^
30,549	M252V, T256A, L309G, H433S, N434W, H435K	2.00 × 10^−7^
30,891	R255S, P257N, H285R, L309D, M428I, N434Y	2.26 × 10^−7^
30,228	M252I, I253S, N434W, H435P, Y436K	2.27 × 10^−7^
30,911	I253T, N286Q, V308P, Q311A, N434Y, Y436S	2.31 × 10^−7^
30,386	T256N, N286L, K288P, T307P, Q311A, M428L, N434Y	2.50 × 10^−7^
30,605	M252E, P257T, H285N, V308P, Q311L, N434Y	2.62 × 10^−7^
30,150	P257Y, Q311T, M428L, N434Y, H435P	2.76 × 10^−7^
30,924	P257A, K288T, T307F, Q311V, N434H, H435K	2.79 × 10^−7^
